# In Vitro Maturation in Women with vs. without Polycystic Ovarian Syndrome: A Systematic Review and Meta-Analysis

**DOI:** 10.1371/journal.pone.0134696

**Published:** 2015-08-04

**Authors:** Charalampos Siristatidis, Theodoros N. Sergentanis, Paraskevi Vogiatzi, Prodromos Kanavidis, Charalampos Chrelias, Nikolaos Papantoniou, Theodora Psaltopoulou

**Affiliations:** 1 Assisted Reproduction Unit, 3rd Department of Obstetrics and Gynecology, University of Athens, Athens, Greece; 2 Department of Hygiene, Epidemiology and Medical Statistics, University of Athens Medical School, Athens, Greece; 3 3rd Department of Obstetrics and Gynecology, University of Athens, Athens, Greece; Women’s Hospital, School of Medicine, Zhejiang University, China. 310006, CHINA

## Abstract

**Objective:**

To evaluate in vitro maturation (IVM) in sub-fertile women with polycystic ovarian syndrome (PCOS) undergoing in vitro fertilisation (IVF), by comparing outcomes with a control group of non-PCOS.

**Study design:**

A search strategy was developed for PubMed and studies reporting rates of the following outcomes (live birth; clinical pregnancy; implantation; cycle cancellation; oocyte maturation; oocyte fertilization; miscarriage) between patients with PCOS, PCO and controls undergoing IVM were deemed eligible. The review was conducted in accordance to the PRISMA guidelines and included studies quality was assessed through the Newcastle-Ottawa Quality scale. ORs with their corresponding 95% CIs were calculated for the main analysis and subgroup analyses were performed for PCOS cases vs. controls and PCOS vs. PCO cases. Alternative analyses were performed for live birth and clinical pregnancy, based on cycles and on women. Subgroup analyses for FSH stimulation, hCG priming and type of procedure (IVF/ICSI) were undertaken for all meta-analyses encompassing at least four study arms. Random effects models were used to calculate pooled effect estimates.

**Results:**

Eleven studies were identified. A total of 268 PCOS patients (328 cycles), 100 PCO patients (110 cycles) and 440 controls (480 cycles) were included in the meta-analysis. A borderline trend towards higher birth rates among PCOS patients emerged (pooled OR = 1.74, 95%CI: 0.99–3.04) mainly reflected at the subgroup analysis vs. controls. Clinical pregnancy (pooled OR = 2.37, 95%CI: 1.53–3.68) and implantation rates (pooled OR = 1.73, 95%CI: 1.06–2.81) were higher, while cancellation rates lower (pooled OR = 0.18, 95%CI: 0.06-0.47) among PCOS vs. non-PCOS subjects; maturation and miscarriage rates did not differ between groups, while a borderline trend towards lower fertilization rates among PCOS patients was observed.

**Conclusion:**

The present meta-analysis provides preliminary evidence on the effectiveness of IVM as a treatment option when offered in sub-fertile PCOS women, as the latter present at least as high outcome rates as those in non-PCOS.

## Introduction

Polycystic ovarian syndrome (PCOS) occurs in 5% to 10% of all women of reproductive age and 50% of women who present with sub-fertility [1]. Clear diagnostic criteria for this condition were identified at the consensus meeting of the European Society of Human Reproduction and Embryology and the American Society for Reproductive Medicine [2]. Sub-fertile women with PCOS will usually benefit from conventional treatments, such as lifestyle changes, laparoscopic ovarian drilling or ovulation induction [3–5], but some will ultimately need assisted reproductive techniques, either if they will need or if they wish, such as controlled ovarian hyperstimulation and IVF. In these cases, controlled ovarian hyperstimulation is closely related to high drug costs, need for daily injections and frequent monitoring, whereas it sometimes results in an increased rate of cycle cancellations and potential life threatening complications due to ovarian hyperstimulation syndrome and in the retrieval of immature oocytes, leading to poor fertilization and lower cleavage, pregnancy, and live birth rates compared to the conventional IVF cycles [6], although this has not been confirmed by other studies [7,8]. In addition, ovulation induction is associated with a high risk of multiple pregnancies due to multiple follicular development, so that it has to be individualized and closely monitored [9].

In order to overcome these complications, in vitro maturation (IVM) has been suggested. It involves the in vitro culture of immature oocytes, from the germinal vesicle or the germinal vesicle breakdown stage, in special laboratory conditions until the metaphase II stage, along with the accompanying cytoplasmic maturation, when the oocyte is considered to be completely mature and ready to undergo fertilization [10]. This can be a potentially useful intervention for women with PCOS-related sub-fertility since these oocytes can retain their maturational and developmental competence after their preterm retrieval from the ovaries [11]. IVM has been also successfully used in a wide range of sub-fertile women, such as poor or high responders undergoing IVF who were at risk for ovarian hyperstimulation syndrome, in women who sought an alternative to conventional IVF in terms of drugs or others for fertility preservation or leukemia/cancer, in those with high antral follicle count scores or in those who wish to avoid gonadotrophin therapy, or in those suffering from ovarian resistance to FSH [12–14].

It remains unclear though, whether PCOS itself contributes to a compromised endometrial receptivity and oocyte quality and whether these lead to reduced fertilization and implantation rates observed after conventional assisted reproduction techniques. Thus, these women who are at risk of developing ovarian hyperstimulation syndrome, following a conventional controlled ovarian hyperstimulation regimen, might benefit from earlier retrieval of oocytes followed by IVM; of note, in such cases, the need for drugs is minimal and often negated. The rationale lies to the in vitro replacement of the oocytes maturation instead of within the ovary. In the context of PCOS, IVM is a mild-approach assisted reproduction technique with no reported cases of ovarian hyperstimulation syndrome. Successful fertilization, embryo development, and term pregnancy resulting from IVM oocytes have been reported [11–13,15–20], obtaining a pregnancy rate up to 35–40% [20], with the first report to be published in the mid 60s [21], and the first pregnancy in an anovulatory PCOS woman in 1994 [11].

The concern expressed regarding the safety of the method with respect to the health of the children, through the in vitro culture conditions [22,23], has been doubted by others who have reported normal obstetric and neonatal outcomes in PCOS [13,24]. Further worries have been reported on low pregnancy and live birth rates, as compared to conventional assisted reproduction techniques [16,25]. There are reports to suggest that IVM should be kept aside and performed only in certain cases when no other option is available, such as in sub-fertile cancer patients, as there are other / newer options for controlled ovarian hyperstimulation in women at high risk for ovarian hyperstimulation syndrome [26]. In a recent update of a Cochrane review, the comparison of IVM to conventional IVF treatment was assessed [27]; nevertheless, the potential differential efficacy of IVM in PCOS versus non-PCOS subjects has not been yet addressed, in the context of a meta-analysis.

The rationale for the current systematic review lies on the fact that sub-fertility with PCOS needing IVF compared with sub-fertility from all other causes (including PCO–polycystic ovaries) could yield different results regarding various outcomes, such as cycle cancellation, pregnancy and live birth rates, when treated with IVM. The triad PCOS—PCO—controls may represent a challenging notion from a methodological point of view. Although complex techniques such as network meta-analysis would seem tempting in this context, close examination of the published literature reveals that the existing corpus of evidence comes exclusively from non-randomized studies. Hence, the background assumptions underlying network meta-analysis, encompassing the existence of randomized controlled trials [28] are not fulfilled; consequently, we approached the aforementioned pattern mainly examining the comparison between PCOS vs. non-PCOS subjects, with the latter group consisting of PCO and/or control subjects. Secondly, in an attempt to evaluate the impact of the reference group, we subdivided the PCOS vs. non-PCOS contrast into separate examination of the PCOS vs. controls and PCOS vs. PCO comparisons. We also evaluated the impact of stimulation and/or priming with gonadotrophins.

## Methods of Review

### Search strategy for the identification of studies

A search strategy was developed for PubMed; the algorithm was the following: (Polycystic OR "PCOS" OR "Stein-Leventhal") AND ("in Vitro Maturation" OR "IVM" OR "Reproductive Medicine" OR "Reproductive Techniques, Assisted" OR "Assisted reproduction" OR "In Vitro Fertilisation" OR "In Vitro Fertilization" OR IVF OR "Intracytoplasmic Sperm Injection" OR ICSI OR "Intracytoplasmatic Sperm Injection") with the end-of-search date was set at 15 October 2013. No restrictions pertaining to publication language or study design were adopted. Reference lists of relevant articles were hand-searched for potentially eligible studies (“snowball” procedure), so as to maximize the amount of synthesized evidence. Study authors were contacted for methodological clarifications and provision of missing data.

### Criteria for considering studies for this review

Studies comparing rates of the following seven outcomes (live birth; clinical pregnancy; implantation; cycle cancellation; oocyte maturation; oocyte fertilization; miscarriage) between PCOS patients, PCO patients and control women undergoing IVM were deemed eligible. All study designs were included. No RCTs are anticipated, since the comparison (PCOS vs. non-PCOS) cannot be randomly assigned; both prospective and retrospective studies were included. Case series and case reports, *in vitro* and animal studies, narrative or systematic reviews were excluded. Regarding the latter studies, they have been searched for individual, potentially eligible cohort studies. Trials on ovum recipients were also excluded, as an issue of embryo quality impairment might be introduced.

If multiple publications (overlapping studies) were identified on the same population, the larger study was used for data extraction, but information from all relevant publications was retained, if necessary. Two authors (CS and PV) working independently and blindly to each other performed the selection of eligible studies, whereas consensus with the last author was reached in case of disagreement.

The quality of all included studies was explicitly assessed. This systematic review and meta-analysis was conducted in accordance with the Preferred Reporting Items for Systematic Reviews and Meta-Analyses (PRISMA) guidelines.

As some of the studies predated the publication of European Society of Human Reproduction and Embryology and the American Society for Reproductive Medicine criteria [2], we included those where the authors clearly stated that the study population was labelled as having PCOS, according to the criteria that each study adopted. Similarly, the definitions of PCO (ultrasonographic appearance of polycystic ovaries, as a rule) and controls (sub-fertile patients with other causes of sub-fertility, such as tubal or male factor) were based on those presented in the individual studies.

### Data extraction

Three authors (TNS, CS and TP) designed and pilot-tested an *ad hoc* developed excel sheet for data extraction; consensus and approval was subsequently obtained by the whole authors’ team. The abstracted data included general information (title, author, year, journal, geographical and clinical setting, study period), study characteristics (number of participants, design, examined outcomes), characteristics of participants (age, BMI, definition of PCOS, PCO and controls, duration and type of infertility, matching factors, previous IVF/ICSI treatments), procedural factors (protocol of IVM, oocyte pretreatment, IVM medium and culture conditions), definitions of outcomes, as well as outcome rates among PCOS, PCO cases and controls.

### Assessment of quality of studies and risk of bias

Studies using different protocols for IVM, for example human chorionic gonadotrophin (hCG) or gonadotrophins priming for the maturation of oocytes before oocyte retrieval as well as the IVM medium and culture conditions during in vitro maturation were carefully recorded.

Although our initial purpose was to assess the existence of publication bias using the Egger’s formal statistical test [29], no statistical evaluation was performed given that the number of included studies per outcome was small (less than 10) and that the power of the test is substantially compromised in this context.

Based on extracted data, the evaluation of quality was based on the nine-item Newcastle-Ottawa Quality scale for all the included studies, a widely used tool for the quality assessment of non-randomized studies [30]. With respect to whether the follow-up was long enough for outcomes to occur, the minimum follow-up of the exposed group was set at birth, given that the main outcome, live birth, is easily found and reported at that time. Concerning completeness of follow-up, a cut-off level was set at 10% of women lost during follow-up. The evaluation of the quality of included studies was performed independently by two reviewers (CS and PV).

### Outcome measures

The primary outcome measure was live birth per woman/couple and per cycle. Additional outcome measures included clinical pregnancy per woman/couple (defined as evidence of a fetal heart on ultrasound at seven gestational weeks), cycle cancellation rate (defined as the ratio of the number of cycles cancelled to the number initiated), miscarriage rate (defined as the number of miscarriages divided by the number of clinical pregnancies), oocyte maturation rate (rate of oocytes matured per oocytes retrieved), fertilization rate (rate of oocytes fertilized per oocytes retrieved) ovarian hyperstimulation syndrome, preterm birth and congenital anomalies of the newborn.

### Data synthesis

Based on the frequencies of outcomes among PCOS, PCO cases and controls, odds ratios (ORs) with their corresponding 95% confidence intervals (CIs) were calculated for the comparison PCOS vs. non-PCOS subjects, with the latter group consisting of PCO and/or control subjects. Two subgroup analyses were additionally performed, namely: i. PCOS cases vs. controls, ii. PCOS vs. PCO cases. ORs >1 denoted more frequent outcome among the first component in the *A* vs. *B* comparison (for instance PCOS in the PCOS vs. non-PCOS comparison). Finally, a secondary, supplementary analysis with in the group of non-PCOS women (PCO cases vs. controls) was performed, to uncover any differences therein. In case of a zero cell in the underlying 2x2 contingency table, an appropriate continuity correction (addition of 0.5) was implemented [31].

Regarding live birth and clinical pregnancy, two alternative analyses were performed, namely one based on cycles (cycles-based approach) and another one based on women (women-based approach). Subgroup analyses by stimulation with FSH, as well as by priming with hCG were *a priori* decided upon to be conducted, for all meta-analyses encompassing at least four study arms. Finally, a subgroup analysis by the type of procedure (ICSI or IVF) was performed and only outcomes comprising four or more published studies were presented.

Random effects (DerSimonian-Laird) models [32] were used to calculate pooled effect estimates. Between-study heterogeneity was assessed by using Cochran Q statistic (significance set at 0.1) and by estimating I^2^ [33]. Statistical analysis was performed using STATA Software/SE 13 (STATA Corporation, College Station, TX, USA).

## Results

### Selection and description of included studies

A total of 268 PCOS patients (328 cycles), 100 PCO patients (110 cycles) and 440 controls (480 cycles) were included in the meta-analysis. Responses of authors contacted for the requested data was satisfactory in more than half of cases.

**Fig 1:**
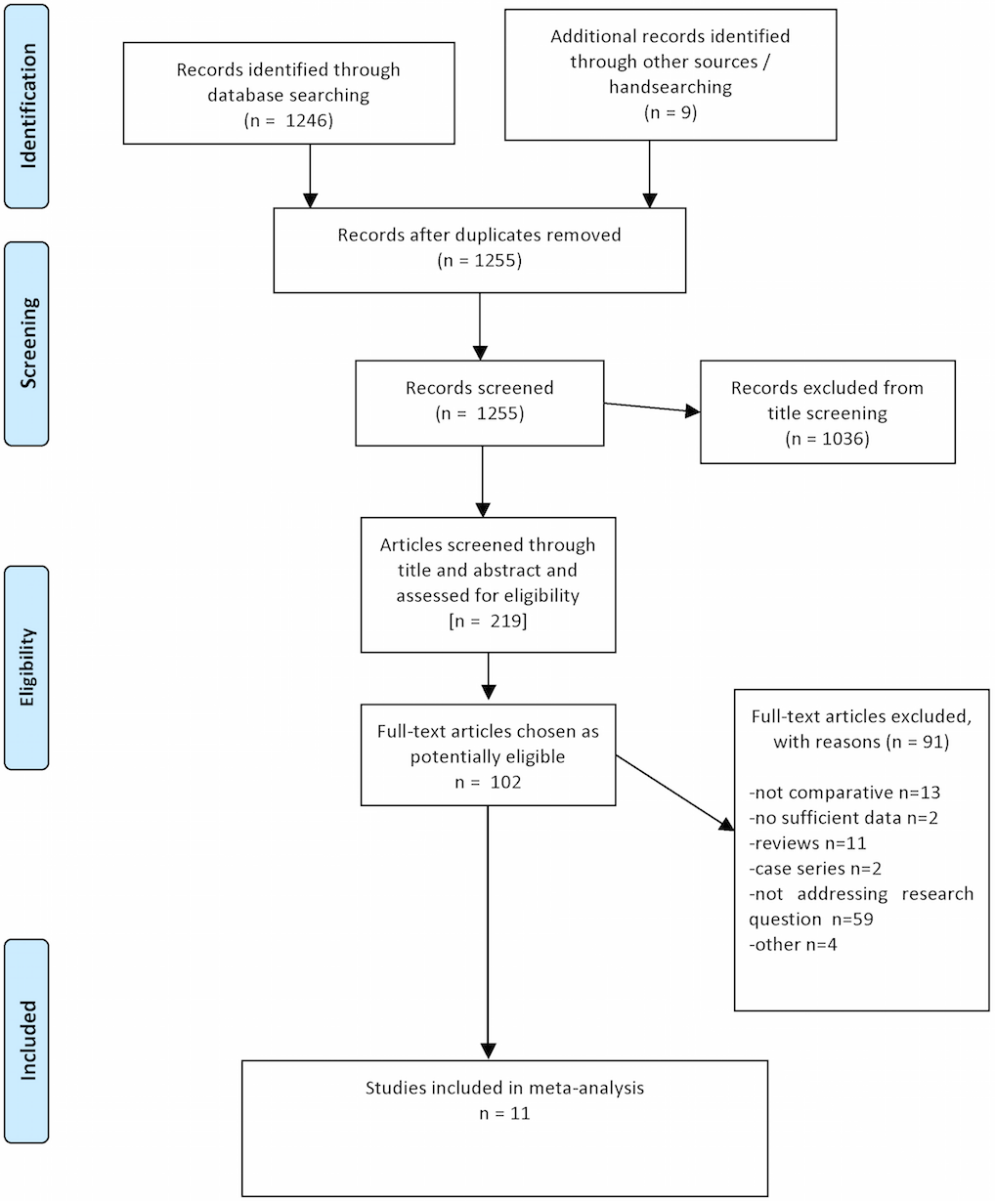
PRISMA Flow Diagram of the studies.

The initial literature search yielded 1255 potentially relevant studies (Fig 1). All titles were carefully checked to exclude irrelevant publications, resulting in 219 potentially eligible studies. The abstracts of these studies were re-examined and eventually 102 manuscripts that provided data to answer the research question were identified. The full text of these studies was examined thoroughly, resulting in the exclusion of 91 (Table A in S2 File). Eventually, 11 studies were considered eligible for inclusion in the meta-analysis [11–13,18,23,34–39]. Characteristics of the studies included are presented in Table 1.

**Table 1:** Characteristics of included studies.

Study (author)	Outcomes examined in the review	Study period	Region	Number of PCOS patients (and cycles)	Number of PCO patients (and cycles)	Number of controls (and cycles)	Matching factors	Mean age (range, y)	BMI (kg/m2)	Duration of infertility (mean, years)	Mean no. of previous IVF/ICSI treatments
Barnes (1996)	Maturation; fertilization	1996 (3 months)	Victoria, Australia	9(9)	N/A	11(11)	None	NR	NR	NR	NR
Buckett (2008)	Miscarriage	1999–2005	Montreal, Quebec, Canada	NR	NR	NR	None	33.4 (20–43)	NR	NR	1.19
Child (2001)	Maturation; fertilization; implantation; clinical pregnancy; miscarriage; live birth; cancellation	1998–2000	Montreal, Quebec, Canada	52 (68)	43(53)	46 (56)	None	33.9 (28–40)	NR	4.7	NR
De Vos (2011)	Maturation; fertilization; implantation; clinical pregnancy; miscarriage; live birth; cancellation	January 2010-October 2010	Brussels, Belgium	34 (60)	5 (5)	N/A	Age and BMI	28.3 (22–36)	22.6 +/- 9.4	NR	NR
Junk (2012)	Maturation; fertilization; clinical pregnancy; miscarriage; live birth; cancellation	NR	Claremont, Australia	47 (47)	19 (19)	N/A	None	31.5 (</ = 42)	24.4	2.45	0.82
Kedem (2013a)	Maturation; fertilization;	2008–2010	Ramat Gan, Israel	20 (30)	N/A	80 (104)	Age and BMI	31 (21–39)	22.2	NR	NR
Mikkelsen (2001)	Maturation; fertilization; clinical pregnancy	1999–2000	Copenhagen, Denmark	29 (29)	N/A	71 (71)	Age and BMI	NR (18–37)	18–29	NR	NR
Soderstrom-Anttila (2005)	Maturation; fertilization; clinical pregnancy; miscarriage; live birth; cancellation	1999–2004	Helsinki, Finland	28 (28)	20 (20)	191 (191)	None	31.7 (<38)	22.0	3.7	0.42
Trounson, experiment 2 (1994)	Maturation; fertilization; clinical pregnancy;	NR	Victoria, Austalia	10 (10)	13 (13)	N/A	None	NR	NR	NR	NR
Vieira (2011)	Maturation	2006–2007	Sao Paolo, Brazil	13 (13)	N/A	27 (27)	None	34.3 (≤38)	23.9	NR	NR
Zhao (2006)	clinical pregnancy; cancellation;	2004–2005	Wenzhou, China	26 (34)	N/A	14 (20)	Age	29 (24–37)	NR	4.8	NR

N/A: Not Applicable; NR: Not Reported

Definition of PCOS according to the European Society of Human Reproduction and Embryology and the American Society for Reproductive Medicine criteria was given in 7 out of 11 studies. In the remaining four reports [11,12,34,38], conducted before 2003, these criteria had not been established yet; nevertheless, the definitions adopted therein were largely compatible with the rationale of European Society of Human Reproduction and Embryology and the American Society for Reproductive Medicine criteria (Table 2). The two largest studies published so far investigating the research question were by Child et al and Junk et al conducted in 2001 and 2012, respectively [12,36], involving 99 PCOS patients and 108 controls in total. The comparison group consisted of PCO women in five studies [11–13,34,36] and in eight of women/couples suffering from other causes of sub-fertility [12,13,18,23,34,37–39].

**Table 2:** Description of cycle and outcome parameters.

Study (author)	Type of infertility	Ascertainment and definition of PCOS	Ascertainment and definition of PCO	Description of the control group	Protocol of IVM (priming)	Oocyte pretreatment	IVM medium and culture conditions	Definition of each outcome
Barnes (1996)	PCOS	Polycystic ovaries with >10 follicles of 2–10 mm, irregular or anovulatory, and/or elevated androgen and LH levels	N/A	Regular menstrual cycles (26–35 days)	No priming. Oocyte recovery at day 9–15 in the control group cycle and when convenient at PCOS group.	HEPES buffered human culture medium,Dulbecco's PBS with 2% heat inactivated fetal calf serum, 0.05 mg/ml penicillin and 0.075 mg/ml streptomycin	TCM-199 with 10% fetal calf serum, 0.075 IU rFSH, 0.5 IU hCG, 0.29 mM pyruvate, 0.05 mg/ml penicillin and 0.075 mg/ml streptomycin for 36–48 hrs	Oocyte maturation: extrusion of the first polar body (MII stage) after 36- 48h in culture; Oocyte fertilization: assessed 14 hours after insemination for the appearance of pronuclei
Buckett (2008)	IVM: PCOS (80%), Male (13%), Unexplained (6%), Tubal (1%); IVF: PCOS (8%), Male (11%), Unexplained (27%), Tubal (29%), Endom (12%), Poor Ovarian reverve (8%), other (5%); ICSI: Male (80%), Unexplained (10%), other (5%), PGD (4%), Endom (1%)	ESHRE/ASRM classification	NR	Other causes of subfertility	10,000IU hCG	Vitrolife IVF medium	IVM Medium supplemented with 75mIU/ml FSH and LH for 24–48 hrs	Clinical miscarriage: pregnancy loss after ultrasonographic evidence of intrauterine pregnancy—gestational sac or fetal heartbeat; Late fetal loss: pregnancy loss after 12 weeks of gestation-including termination after congenital abnormality
Child (2001)	PCOS, PCO	Polycystic ovaries, oligomenorrhea or amenorhea and/or clinical or biochemical evidence of hyperandrogenism	Polycystic ovaries with > = 10 small (2–8 mm) cysts in early follicular phase	Women without polycystic ovaries on transvaginal ultrasound	10,000 IU of hCG	0.9% saline with 2IU/L heparin	TC-199 medium with 20% heat-inactivated maternal serum, 25 mol/Lpyruvic acid and 75 mIU/mL FSH + LH	Oocyte maturation: extrusion of the first polar body (MII stage) 24–48 hours postcollection; Fertilization: appearance of two distinct pronuclei and two polar bodies 18 hours after ICSI; Implantation rate: calculated by dividing the total number of gestational sacs by the total number of embryos transferred within a treatment group; Clinical pregnancy: presence of a gestational sac by transvaginal ultrasound 4 weeks after oocyte retrieval; Miscarriage: NR
De Vos (2011)	PCOS, PCO	Rotterdam criteria	Ultrasound appearance of multiple cysts and regular cycles	N/A	PCOS and PCO: 150IU/day hMG or FSH starting on Day 3 for 3 days. No hCG trigger.	No	Medicult IVM System (Origio)	Oocyte maturation extrusion of the first polar body (MII stage) after 40h in culture; Fertilization: NR; Implantation rate: ratio between the number of gestational sacs and the total number of embryos transfered; Clinical pregnancy: presence of intrauterine gestational sac by transvaginal ultrasound scan >/ = 5 weeks after ET; Miscarriage: NR
Junk (2012)	PCOS, PCO	Rotterdam criteria, ESHRE/ASRM classification	Polycystic ovaries confirmed by ultrasound assessment	N/A	FSH priming 100IU or 150IU for 3 days,	NR	G2 Plus medium with 10% of patient's heat-inactivated serum, 0.1IU/ml FSH and 0.5IU/ml of hCG for 24-26hrs	Oocyte maturation: extrusion of thefirst polar body (MII stage); Fertilization: resence of pronuclei 16-18hrs after ICSI; Clinical pregnancy: heartbeat detectable at pelvic ultrasound at 7wks from last menstrual period; Miscarriage: NR;
Kedem (2013a)	PCOS, Control group NR	Rotterdam criteria	N/A	Regular menstrual cycles, morphologically and endocrinologically normal ovaries	150IU/day rFSH starting on Day 3 for 3 days. 10,000 IU hCG	No	IVM medium (SAGE) supplemented with 75IU FSH and 75IU LH	Oocyte maturation: extrusion of the first polar body (MII stage) after 24-48h in culture; Fertilization: presence of pronuclei 18 h after ICSI;
Mikkelsen (2001)	PCOS, Control group: male factor (n = 32), tubal factor (n = 34) or combination (n = 5)	Polycystic ovaries defined as >10 follicles 2-8mm in diameter, oligomenorrhoea or amenorrhoea for >6months, endocrine abnormalities with elevated LH/FSH ratio or elevated androgens	N/A	Regular menstrual cycles with normal ovaries and either male factor (n = 32), tubal factor (n = 34) or a combination of these factors (n = 5)	No reported priming in control group. PCOS group received rFSH 150IU/Day on Day 3 for 3 days. No hCG priming.	Ham F-10 with heparin	TCM -199 with 0.3mmol/l Na pyruvate, 1500 IU/ml penicillin G, 50 mg/ml sreptomycin sulphate, 1μγ/ml oestradiol, rec-FSH 0.075 IU/ml, 0.5 IU/ml HCG and patient serum, for 28–30 hrs	Oocyte maturation: extrusion of the first polar body (MII stage); Fertilization: two distinct pronuclei and two polar bodies at 18–20 hours after ICSI; Clinical pregnancy: presence of a gestational sac with evidence of heart activity by transvaginal ultrasound 5 weeks after ET;
Soderstrom-Anttila (2005)	Regular IVF: tubal (15), other female (10), male (21), multiple (14), unexplained (31); regular ICSI: tubal (9), other female (6), male (51), multiple (22), unexplained (12); PCO IVF: other female (6), male (1), multiple (5), unexplained (1), PCO ICSI: other female (2), male (1), multiple (4); PCOS IVF: other female (8), male (1), multiple (8), unexplained (1), PCOS ICSI: other female (1), multiple (9)	Rotterdam ESHRE/ASRM classification	Patients with PCO ovaries on ultrasound scan and follicular selection	Normal ovaries and regular cycles	10,000IU HCG	Dulbecco's Phosphate Buffered Saline (PBS) containing heparin	TCM-199 with 10% patient serum, 0.075IU/ml rec-FSH, 0.1IU/ml hCG, pyruvate, penicillin and streptomycin OR Medicult IVM Medium supplemented with 10% patient serum, 0.075IU/ml rec-FSH, 0.1IU/ml hCG for 24–36 hrs	Oocyte maturation: extrusion of the first polar body (MII stage) at 30-48hrs postcollection; Fertilization: NR; Implantation: positive serum hCG level; Clinical pregnancy: presence of a gestational sac or fetal by transvaginal ultrasound at 5 weeks after ET; Miscarriage: NR;
Trounson, experiment 2 (1994)	NR	Elevated androgen levels, LH:FSH ratios>2, and/or hirsutism, increased body weight	Ultrasound appearance of multiple cysts	Normal ovarian morphology	No priming	NR	EMEM with and without 0.5IU/ml hCG and mature GC coculture solution- TCM-199 containing 10% FCS, 0.075IU/ml hMG, 1mg/ml E2 with and without 0.5IU/ml hCG for 29.5 to 32.5hrs	Oocyte maturation: extrusion of the first polar body (MII stage); Fertilization: presence of two pronuclei (2PN); Clinical pregnancy: NR;
Vieira (2011)	PCOS: chronic annovulation, Control: male infertility and/or tubal factor	Rotterdam criteria	N/A	Normal ovarian morphology and no pelvic disease	200-300IU FSH for 6 days. 250μg hCG,			Oocyte maturation: extrusion of the first polar body (MII stage)
Zhao (2006)	PCOS, previous ART/IVF failures	Rotterdam ESHRE/ASRM classification	N/A	Previous ART failures	No priming	NR	TCM 199 supplemented with 0.075 IU FSH, 0.1 IU hCG for 24–48 hours	NR

Footnotes:

N/A: Not Applicable; NR: Not Reported

IVF: In Vitro Fertilisation; ICSI: Intracytoplasmic sperm injection; PGD: Pre-implantation genetic diagnosis; ART: Assisted reproductive technology; ESHRE/ASRM: European Society of Human Reproduction and Embryology (ESHRE) and the American Society for Reproductive Medicine (ASRM); HEPES buffer: N-(2-hydroxyethyl) piperazine-N'-2-ethanesulfonic acid; TCM 199: tissue culture medium 199; EMEM: Eagle's minimal essential medium; LH: Luteinizing hormone; FSH: Follicle stimulating hormone; HCG: Human chorionic gonadotropin; ET: Embryo transfer.

HCG was used to trigger final oocyte maturation in five studies [12,13,23,37,39], mild stimulation with FSH was used in five studies [35–39], while in three no hormonal priming was used [11,18,34] (Table 2). No studies reported on ovarian hyperstimulation syndrome and preterm birth, whereas one study reported on congenital abnormalities [23].

### Synthesis of studies

**Fig 2:**
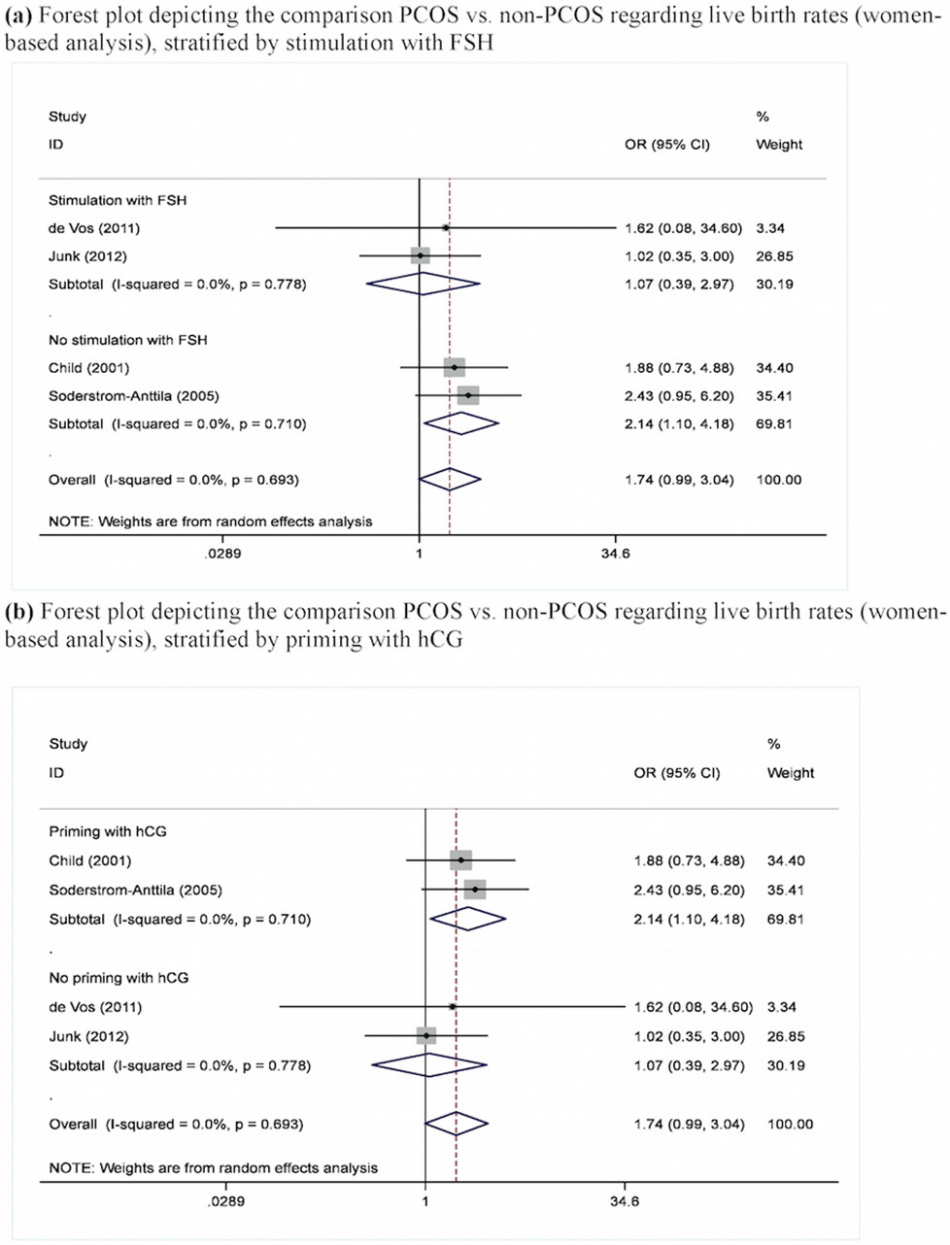
(a). Comparison PCOS vs. non-PCOS regarding live birth rates (women-based analysis), stratified by stimulation with FSH. (b) Comparison PCOS vs. non-PCOS regarding live birth rates (women-based analysis), stratified by priming with hCG.

Table 3 presents the results of the meta-analysis, by outcome and comparison group. Live birth rates did not differ between PCOS and non-PCOS subjects, at the cycles-based analysis (Figures A-C in S1 File); however, at the women-based analyses (Fig 2 and Figures B, D in S1 File), a borderline trend towards higher birth rates among PCOS patients emerged (pooled OR = 1.74, 95%CI: 0.99–3.04, p = 0.053, Fig 2), mainly reflected at the subgroup analysis vs. controls (pooled OR = 3.72, 95%, CI: 0.95–14.49, p = 0.059, Figure Bb in S1 File).

**Table 3:** Results of the meta-analyses addressing the three comparisons (PCOS vs. controls; PCO vs. controls; PCOS vs. PCO) in the examined outcomes (Bold cells denote statistically significant associations).

	PCOS vs. non-PCOS (PCO/controls)			Subgroup analysis: PCOS vs. controls			Subgroup analysis: PCOS vs. PCO		
	n^§^	OR (95%CI)	Heterogeneity I^2^, p	n^§^	OR (95%CI)	Heterogeneity I^2^, p	n^§^	OR (95%CI)	Heterogeneity I^2^, p
Live birth (cycles-based analysis)	4	1.56 (0.90–2.72)	0.0%, 0.766	2	3.27 (0.81–13.28)	42.6%, 0.187	4	1.09 (0.57–2.10)	0.0%, 0.643
Live birth (women-based analysis)	4	1.74 (0.99–3.04)	0.0%, 0.693	2	3.72 (0.95–14.49)	39.8%, 0.197	4	1.18 (0.61–2.29)	0.0%, 0.604
Clinical pregnancy (cycles-based analysis)	7	**2.23 (1.45–3.43)**	0.0%, 0.722	4	**3.09 (1.46–6.53)**	36.8%, 0.191	5	1.60 (0.89–2.88)	0.0%, 0.552
Clinical pregnancy (women-based analysis)	7	**2.37 (1.53–3.68)**	0.0%, 0.666	4	**3.29 (1.42–7.62)**	46.8%, 0.131	5	1.75 (0.96–3.20)	0.0%, 0.586
Implantation (embryos-based analysis)	3	**1.73 (1.06–2.81)**	0.0%, 0.856	2	2.89 (0.75–11.08)	64.2%, 0.095	3	1.45 (0.62–3.39)	19.2%, 0.290
Cancellation (cycles-based analysis)	5	**0.18 (0.06–0.47)**	0.0%, 0.895	3	**0.15 (0.05–0.44)**	0.0%, 0.659	4	**0.25 (0.07–0.92)**	0.0%, 0.783
Maturation (oocytes-based analysis)	8	0.87 (0.70–1.08)	63.7%, 0.007	5	**0.74 (0.59–0.93)**	52.4%, 0.078	5	1.03 (0.88–1.21)	0.0%, 0.734
Fertilization (oocytes-based analysis)	8	0.78 (0.60–1.03)	71.3%, 0.001	5	0.76 (0.55–1.04)	72.9%, 0.005	5	0.88 (0.54–1.43)	79.9%, 0.001
Miscarriage (women-based analysis)	4	1.51 (0.72–3.17)	0.0%, 0.795	3	1.22 (0.52–2.85)	0.0%, 0.847	3	2.60 (0.71–9.54)	0.0%, 0.879

^§^number of study arms

**Fig 3:**
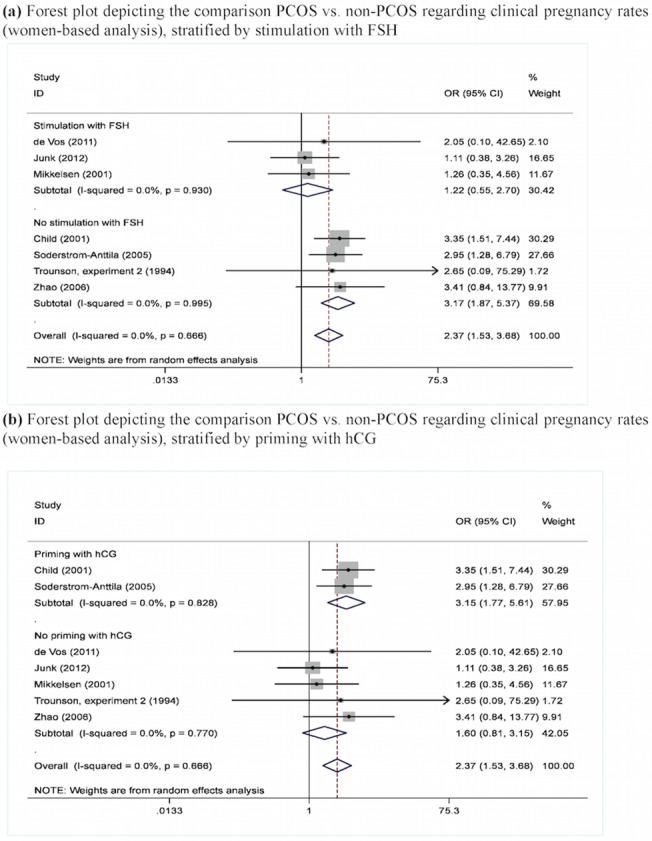
(a). Comparison PCOS vs. non-PCOS regarding clinical pregnancy rates (women-based analysis), stratified by stimulation with FSH. (b) Comparison PCOS vs. non-PCOS regarding clinical pregnancy rates (women-based analysis), stratified by priming with hCG.

Regarding clinical pregnancy (Fig 3 and Figures E-I in S1 File), higher rates were noted between PCOS and non-PCOS subjects both at the cycles-based analysis (pooled OR = 2.23, 95%CI: 1.45–3.43, Figure E in S1 File) and the women-based approach (pooled OR = 2.37, 95%CI: 1.53–3.68, Fig 3). Once again, these associations were reproduced upon the analysis versus controls (pooled OR = 3.09, 95%CI: 1.46–6.53, Figure F in S1 File) and (pooled OR = 3.29, 95%CI: 1.42–7.62, Figure H in S1 File). Accordingly, implantation rates were higher in PCOS versus non-PCOS subjects (pooled OR = 1.73, 95%CI: 1.06–2.81, Figure J in S1 File); nevertheless, statistical significance was not reached at the subgroup analyses despite the sizeable effect estimates, possibly due to the smaller number of study arms and included cases (Figure K in S1 File).

Cancellation rates (Figures K-M in S1 File) were lower among PCOS patients (pooled OR = 0.18, 95%CI: 0.06–0.47, Figure L in S1 File), with this pattern replicated in both subgroup analyses (pooled OR = 0.15, 95%CI: 0.05–0.44 vs. controls, Figure M in S1 File; pooled OR = 0.25, 95%CI: 0.07–0.92 vs. PCO subjects, Figure N in S1 File).

Maturation rates (Figures O-Q in S1 File) did not differ between the compared groups, with the exception of lower rates among PCOS patients vs. controls (pooled OR = 0.74, 95%CI: 0.59–0.93, Figure P in S1 File).

Regarding fertilization rates (Figures R-T in S1 File), a borderline trend towards lower rates among PCOS patients was observed (pooled OR = 0.78, 95%CI: 0.60–1.03, p = 0.080, vs. non-PCOS, Figure R in S1 File; pooled OR = 0.76, 95%CI: 0.55–1.04, p = 0.089, vs. controls, Figure S in S1 File). Miscarriage rates did not differ between the examined groups (Figures U,V in S1 File). Table B in S2 File presents the secondary analysis within the non-PCOS women group (PCO vs. control women); no statistically significant differences were noted therein (Figures W-AC in S1 File), but only two studies provided the relevant data.

### Stratification by stimulation with FSH, priming with hCG and type of procedure (IVF/ICSI)

Regarding subgroup analyses by stimulation with FSH and priming with hCG (Table C in S2 File), the statistically significant findings tended to be replicated upon the subgroups with the larger number of study arms, highlighting the loss of statistical power along with smaller subgroups. Of note, however, was the marked persistence of PCOS superiority in terms of clinical pregnancy rates among the two studies undertaking priming with hCG both at the cycles-based (pooled OR = 2.88, 95%CI: 1.64–5.07, Figure Eb in S1 File) and women-based analyses (pooled OR = 3.15, 95%CI: 1.77–5.61, Fig 3B), contrary to the null pattern in the five studies skipping out any priming with hCG.

Subgroup analyses by the type of procedure (ICSI or IVF) are presented in Table D (in S2 File). Although subgroup analyses were often hampered by the small number of study arms, the better performance of PCOS subjects vs. non-PCOS tended to be more sizable in studies adopting IVF (Figures AD-AG in S1 File).

### Quality of the included studies

Rating of the quality of studies according to the Newcastle-Ottawa score is presented in Table E in S2 File. Quality scores ranged between 5 and 9. As expected, older studies [11,34,38] tended to receive a lower score, as compared to the newer ones.

## Conclusions

The performed meta-analysis provided evidence demonstrating that IVM seems to be a preferable approach in treating women with PCOS during an IVF cycle as compared to those without the syndrome. Based on 11 trials with 268 PCOS, 100 PCO patients and 440 women with other causes of sub-fertility, we conclude that IVM appears to be a more efficient treatment option in terms of clinical pregnancy, implantation and cycle cancellation rates in women with PCOS when compared to the non-PCOS group; importantly, we also observed a borderline but meaningful trend in live birth rates in the PCOS group, favoring IVM.

Towards investigation of our research question, we compared IVM outcome on specific parameters, for populations with and without PCOS, including controls (normal ovarian function) and women with PCO. Women with PCOS comprise an ideal target group for IVM, since they present with greater number of antral follicles per ovary, as compared with non-PCOS and importantly, these are more likely to experience ovarian hyperstimulation syndrome. Unexpectedly, our initial search revealed that the majority of studies investigating IVM in sub-fertile PCOS women were not comparative [27]. A number of these studies pointed to similar outcomes for women with and without PCOS undergoing IVM, but without performing formal comparisons [16,22].

Our meta-analysis suggests a relative preponderance of the PCOS group regarding the primary outcome in women-based analysis, reported in four [12,13,35,36] out of the 11 included studies. This finding opposes to the reported–and possibly expected- results so far [40]. Equally important is the significant difference observed in the secondary outcomes of this study (pregnancy, implantation and cycle cancellation rates), reported in seven [11–13,18,35,36,38], three [12,13,35] and six [11–13,18,35,36] included studies, respectively. Moreover, maturation rates, between PCOS and non-PCOS groups, as reported in eight studies [11–13,34–37,39], were similar, apart from the comparison of PCOS with the control population, where proved inferior, a finding that was rather expected for PCOS patients. Adversely, there was a trend towards lower fertilization rates (8/11 studies) [11–13,34–38], in PCOS patients, although this was not observed in miscarriage rates (4/11 studies) [12,13,23,36].

In addition, no differences were observed between women with PCO and women with normal ovarian function, demonstrating that both groups share the same results among the non-PCOS populations in terms of IVM outcome. Results have been previously reported, emphasizing the need for further investigation of the effect of gonadotrophin priming in IVM, both in a clinical and an embryology laboratory setting [40]; of note, the number of included study arms in the subgroup analyses pertaining to either gonadotrophin stimulation or priming was limited in our approach, not allowing reaching safe conclusions. Similarly, in the subgroup analysis IVF versus ICSI, the better performance of PCOS vs. non-PCOS tended to be more sizable in studies adopting IVF, probably denoting an “interventional” negative effect of ICSI on the immature oocyte; however, the small number of study arms does not allow firm conclusions to be drawn.

During IVF, women with PCOS have theoretically an increased risk of cycle cancellation and developing ovarian hyperstimulation syndrome, while usually a poorer outcome is expected. Despite this, previous data has presented similar pregnancy and live birth rates per cycle, although cancellation rates tend to be higher, as opposed to the lowered fertilization rates [7]. Elevated E_2_, LH and androgen levels appear to play a key role, as these possibly exert a detrimental effect in oocyte maturation and embryonic development [27]. During IVM, ovarian stimulation is minimal or even absent and this partially explains the findings of the current review.

The remainder outcome parameters (ovarian hyperstimulation syndrome and preterm birth) initially included in our search strategy, were not reported in any of the comparative studies included in our review. The presence of congenital anomalies, following birth, was only reported in one study [23] and therefore no definite conclusions could be drawn.

No significant statistical heterogeneity was observed in the majority of the performed analyses. Accordingly, the PCOS group was clearly described even in the four studies conducted before the establishment of the European Society of Human Reproduction and Embryology and the American Society for Reproductive Medicine criteria, whereas the oocyte retrieval and maturation protocols were similar for all included studies.

The present meta-analysis, despite its originality, bears certain limitations essentially reflecting those of the published literature. First, no RCTs were identified, as the comparison (i.e. PCOS vs. non-PCOS) cannot be randomly assigned; moreover, the sample size of the included studies was rather small, possibly hampering the statistical power in some analyses. For instance, the difference between PCOS and non-PCOS subjects regarding live birth rates was of borderline significance (p = 0.053, women-based analysis); according to our power calculation (data not shown), a sample of 175:350 women would be needed for the optimal power of 90%, whereas our meta-analysis included 161:353 women. This implies that the addition of a slightly larger number of cases may well result in the emergence of formal significance concerning live birth rates. Moreover, the fact that less than 10 study arms were included in the comparisons did not allow the reliable performance of meta-regression analysis, as stated in the Cochrane Handbook for Systematic Reviews of Interventions [33]; therefore, the potential modifying effects mediated by meaningful factors, such as age, could not be assessed. In any case, however, the modifying effect of age seemed rather minimal, as the mean age of included subjects in the eligible studies ranged minimally, namely between 28.3 [35] and 34.3 years [39]. Furthermore, the eligible studies corresponded to 268 PCOS patients, 100 PCO subjects and 440 controls, namely to groups with unequal numbers, whereas the results would ideally have been based on almost equally sized treatment groups. In addition, the variability in the definition of PCOS might be considered as a potential source of heterogeneity, as the European Society of Human Reproduction and Embryology and the American Society for Reproductive Medicine criteria were adopted in seven out of 11 studies; nevertheless, the criteria in the remaining four reports [11,12,34,38] are largely compatible with this rationale. Finally, given the variable clinical settings and countries in which the eligible studies were performed, it would seem desirable to anticipate a future, large clinical trial on comparable PCOS, PCO and control subjects with strict follow-up and documentation of outcomes, aiming to further validate the clinical relevance of previously published results which this meta-analysis succinctly summarized.

For the complete evaluation of the effectiveness of IVM in PCOS, further parameters should be taken into consideration. Importantly, the safety of IVM should be efficiently evaluated by including long-term findings (>10 years follow-up) to examine potential developmental and undiscovered congenital anomalies of the offspring, while the cost of the modality for both individuals and national health system/insurance bodies should be evaluated. Finally, properly conducted prospective studies with larger samples are required in order to confirm the findings of the current review.

## Supporting Information

S1 File Forest plot depicting the comparison PCOS vs. non-PCOS regarding live birth rates (cycles-based analysis), stratified by (a) stimulation with FSH, (b) priming with hCG (Figure A). Forest plot depicting the subgroup analysis PCOS vs. controls regarding live birth rates. (a) cycles-based analysis; (b) women-based analysis (**Figure B**). Forest plot depicting the subgroup analysis PCOS vs. PCO regarding live birth rates (cycles-based analysis), stratified by (a) stimulation with FSH, (b) priming with hCG (**Figure C**). Forest plot depicting the subgroup analysis PCOS vs. PCO regarding live birth rates (women-based analysis), stratified by (a) stimulation with FSH, (b) priming with hCG (**Figure D**). Forest plot depicting the comparison PCOS vs. non-PCOS regarding clinical pregnancy rates (cycles-based analysis), stratified by (a) stimulation with FSH, (b) priming with hCG (**Figure E**). Forest plot depicting the subgroup analysis PCOS vs. healthy controls regarding clinical pregnancy rates. (cycles-based analysis), stratified by (a) stimulation with FSH, (b) priming with hCG (**Figure F**). Forest plot depicting the subgroup analysis PCOS vs. PCO regarding clinical pregnancy rates. (cycles-based analysis), stratified by (a) stimulation with FSH, (b) priming with hCG **(Figure G)**. Forest plot depicting the subgroup analysis PCOS vs. controls regarding clinical pregnancy rates. (women-based analysis), stratified by (a) stimulation with FSH, (b) priming with hCG (**Figure H**). Forest plot depicting the subgroup analysis PCOS vs. PCO regarding clinical pregnancy rates. (women-based analysis), stratified by (a) stimulation with FSH, (b) priming with hCG (**Figure I**). Forest plot depicting the comparison PCOS vs. non-PCOS regarding implantation rates (embryos-based analysis) (**Figure J**). Forest plot depicting the subgroup analyses regarding implantation rates (embryos-based analysis). (a) PCOS vs. healthy controls, (b) PCOS vs. PCO (**Figure K**). Forest plot depicting the comparison PCOS vs. non-PCOS regarding cancellation rates (cycles-based analysis), stratified by (a) stimulation with FSH, (b) priming with hCG (**Figure L**). Forest plot depicting the subgroup analysis PCOS vs. controls regarding cancellation rates (cycles-based analysis) (**Figure M**). Forest plot depicting the subgroup analysis PCOS vs. PCO regarding cancellation rates (cycles-based analysis), stratified by (a) stimulation with FSH, (b) priming with hCG (**Figure N**). Forest plot depicting the comparison PCOS vs. non-PCOS regarding maturation rates (oocytes-based analysis), stratified by (a) stimulation with FSH, (b) priming with hCG (**Figure O**). Forest plot depicting the subgroup analysis PCOS vs. controls regarding maturation rates (oocytes-based analysis), stratified by (a) stimulation with FSH, (b) priming with hCG (**Figure P**). Forest plot depicting the subgroup analysis PCOS vs. PCO regarding maturation rates (oocytes-based analysis), stratified by (a) stimulation with FSH, (b) priming with hCG (**Figure Q**). Forest plot depicting the comparison PCOS vs. non-PCOS regarding fertilization rates (oocytes-based analysis), stratified by (a) stimulation with FSH, (b) priming with hCG (**Figure R**). Forest plot depicting the subgroup analysis PCOS vs. controls regarding fertilization rates (oocytes-based analysis), stratified by (a) stimulation with FSH, (b) priming with hCG (**Figure S**). Forest plot depicting the subgroup analysis PCOS vs. PCO regarding fertilization rates (oocytes-based analysis), stratified by (a) stimulation with FSH, (b) priming with hCG (**Figure T**). Forest plot depicting the comparison PCOS vs. non-PCOS regarding miscarriage rates (women-based analysis), stratified by (a) stimulation with FSH, (b) priming with hCG (**Figure U**). Forest plot depicting the subgroup analysis regarding miscarriage rates (women-based analysis). (a) PCOS vs. healthy controls, (b) PCOS vs. PCO (**Figure V**). Secondary analysis within the non-PCOS group. Forest plot depicting the comparison PCO vs. controls regarding live birth rates. (a) cycles-based analysis; (b) women-based analysis (**Figure W**). Secondary analysis within the non-PCOS group. Forest plot depicting the comparison PCO vs. controls regarding clinical pregnancy rates. (a) cycles-based analysis; (b) women-based analysis (**Figure X**). Secondary analysis within the non-PCOS group. Forest plot depicting the comparison PCO vs. controls regarding implantation rates (embryos-based analysis) (**Figure Y**). Secondary analysis within the non-PCOS group. Forest plot depicting the comparison PCO vs. controls regarding cancellation rates (cycles-based analysis) (**Figure Z**). Secondary analysis within the non-PCOS group. Forest plot depicting the comparison PCO vs. controls regarding maturation rates (oocytes-based analysis) (**Figure AA**). Secondary analysis within the non-PCOS group. Forest plot depicting the comparison PCO vs. controls regarding fertilization rates (oocytes-based analysis) (**Figure AB**). Secondary analysis within the non-PCOS group. Forest plot depicting the comparison PCO vs. controls regarding miscarriage rates (women-based analysis) (**Figure AC**). Forest plot depicting the comparison PCOS vs. non-PCOS regarding live birth rates, (a): cycles-based analysis, (b): women-based analysis, separately in study arms examining ICSI or IVF (**Figure AD**). Forest plot depicting the comparison PCOS vs. non-PCOS regarding clinical pregnancy rates, (a) cycles-based analysis, (b) women-based analysis, separately in study arms examining ICSI or IVF (**Figure AE**). Forest plot depicting the comparison PCOS vs. non-PCOS regarding (a) cancellation rates (cycles-based analysis), (b) maturation rates (oocytes-based analysis), separately in study arms examining ICSI or IVF (**Figure AF**). Forest plot depicting the comparison PCOS vs. non-PCOS regarding (a) fertilization rates (oocytes-based analysis), (b) miscarriage rates (women-based analysis), separately in study arms examining ICSI or IVF (**Figure AG**).
(DOCX)

S2 File Excluded studies and their references (Table A). Results of the meta-analyses addressing the comparison “PCO vs. controls” (analysis within non-PCOS women) regarding the examined outcomes. Bold cells denote statistically significant associations (**Table B**). Subgroup analyses by stimulation with FSH and priming with hCG, regarding the outcomes comprising four or more study arms; therefore no subgroup analyses are presented regarding implantation, as well as any of the “PCO vs. controls” comparisons. Bold cells denote statistically significant associations (**Table C**). Subgroup analyses by ICSI / IVF procedure; the study by Soderstrom-Anttila, 2005 was subdivided into two separate study arms for this analysis. Only outcomes comprising four or more published studies are presented; bold cells denote statistically significant associations (**Table D**). Evaluation of quality based on the Newcastle-Ottawa scale for all eleven included studies (**Table E**).
(DOCX)
