# Supplementary material for: In Vitro Maturation in Women with vs. without Polycystic Ovarian Syndrome: A Systematic Review and Meta-Analysis
Source: PLoS One. 2015 Aug 4;10(8):e0134696. doi: 10.1371/journal.pone.0134696 (PMC4524709; doi:10.1371/journal.pone.0134696)
Supplement: S1 File — Forest plot depicting the subgroup analysis PCOS vs. controls regarding live birth rates. (a) cycles-based analysis; (b) women-based analysis (Figure B). Forest plot depicting the subgroup analysis PCOS vs. PCO regarding live birth rates (cycles-based analysis), stratified by (a) stimulation with FSH, (b) priming with hCG (Figure C). Forest plot depicting the subgroup analysis PCOS vs. PCO regarding live birth rates (women-based analysis), stratified by (a) stimulation with FSH, (b) priming with hCG (Figure D). Forest plot depicting the comparison PCOS vs. non-PCOS regarding clinical pregnancy rates (cycles-based analysis), stratified by (a) stimulation with FSH, (b) priming with hCG (Figure E). Forest plot depicting the subgroup analysis PCOS vs. healthy controls regarding clinical pregnancy rates. (cycles-based analysis), stratified by (a) stimulation with FSH, (b) priming with hCG (Figure F). Forest plot depicting the subgroup analysis PCOS vs. PCO regarding clinical pregnancy rates. (cycles-based analysis), stratified by (a) stimulation with FSH, (b) priming with hCG (Figure G). Forest plot depicting the subgroup analysis PCOS vs. controls regarding clinical pregnancy rates. (women-based analysis), stratified by (a) stimulation with FSH, (b) priming with hCG (Figure H). Forest plot depicting the subgroup analysis PCOS vs. PCO regarding clinical pregnancy rates. (women-based analysis), stratified by (a) stimulation with FSH, (b) priming with hCG (Figure I). Forest plot depicting the comparison PCOS vs. non-PCOS regarding implantation rates (embryos-based analysis) (Figure J). Forest plot depicting the subgroup analyses regarding implantation rates (embryos-based analysis). (a) PCOS vs. healthy controls, (b) PCOS vs. PCO (Figure K). Forest plot depicting the comparison PCOS vs. non-PCOS regarding cancellation rates (cycles-based analysis), stratified by (a) stimulation with FSH, (b) priming with hCG (Figure L). Forest plot depicting the subgroup analysis PCOS vs. [file pone.0134696.s001.docx]

**Figure A.** Forest plot depicting the comparison PCOS vs. non-PCOS regarding live birth rates (cycles-based analysis), stratified by (a) stimulation with FSH, (b) priming with hCG.

**(a)**

**(b)**

**Figure B.** Forest plot depicting the subgroup analysis PCOS vs. controls regarding live birth rates. (a) cycles-based analysis; (b) women-based analysis.

**(a)**

**(b)**

**Figure C.** Forest plot depicting the subgroup analysis PCOS vs. PCO regarding live birth rates (cycles-based analysis), stratified by (a) stimulation with FSH, (b) priming with hCG.

**(a)**

**(b)**

**Figure D.** Forest plot depicting the subgroup analysis PCOS vs. PCO regarding live birth rates (women-based analysis), stratified by (a) stimulation with FSH, (b) priming with hCG.

**(a)**

**(b)**

**Figure E.** Forest plot depicting the comparison PCOS vs. non-PCOS regarding clinical pregnancy rates (cycles-based analysis), stratified by (a) stimulation with FSH, (b) priming with hCG.

**(a)**

**(b)**

**Figure F.** Forest plot depicting the subgroup analysis PCOS vs. healthy controls regarding clinical pregnancy rates. (cycles-based analysis), stratified by (a) stimulation with FSH, (b) priming with hCG.

**(a)**

**(b)**

**Figure G.** Forest plot depicting the subgroup analysis PCOS vs. PCO regarding clinical pregnancy rates. (cycles-based analysis), stratified by (a) stimulation with FSH, (b) priming with hCG.

**(a)**

**(b)**

**Figure H.** Forest plot depicting the subgroup analysis PCOS vs. controls regarding clinical pregnancy rates. (women-based analysis), stratified by (a) stimulation with FSH, (b) priming with hCG.

**(a)**

**(b)**

**Figure I.** Forest plot depicting the subgroup analysis PCOS vs. PCO regarding clinical pregnancy rates. (women-based analysis), stratified by (a) stimulation with FSH, (b) priming with hCG.

**(a)**

**(b)**

**Figure J.** Forest plot depicting the comparison PCOS vs. non-PCOS regarding implantation rates (embryos-based analysis).

**Figure K.** Forest plot depicting the subgroup analyses regarding implantation rates (embryos-based analysis). (a) PCOS vs. healthy controls, (b) PCOS vs. PCO

**(a)**

**(b)**

**Figure L.** Forest plot depicting the comparison PCOS vs. non-PCOS regarding cancellation rates (cycles-based analysis), stratified by (a) stimulation with FSH, (b) priming with hCG.

**(a)**

**(b)**

**Figure M.** Forest plot depicting the subgroup analysis PCOS vs. controls regarding cancellation rates (cycles-based analysis).

**Figure N.** Forest plot depicting the subgroup analysis PCOS vs. PCO regarding cancellation rates (cycles-based analysis), stratified by (a) stimulation with FSH, (b) priming with hCG.

**(a)**

**(b)**

**Figure O.** Forest plot depicting the comparison PCOS vs. non-PCOS regarding maturation rates (oocytes-based analysis), stratified by (a) stimulation with FSH, (b) priming with hCG.

**(a)**

**(b)**

**Figure P.** Forest plot depicting the subgroup analysis PCOS vs. controls regarding maturation rates (oocytes-based analysis), stratified by (a) stimulation with FSH, (b) priming with hCG.

**(a)**

**(b)**

**Figure Q.** Forest plot depicting the subgroup analysis PCOS vs. PCO regarding maturation rates (oocytes-based analysis), stratified by (a) stimulation with FSH, (b) priming with hCG.

**(a)**

**(b)**

**Figure R.** Forest plot depicting the comparison PCOS vs. non-PCOS regarding fertilization rates (oocytes-based analysis), stratified by (a) stimulation with FSH, (b) priming with hCG.

**(a)**

**(b)**

**Figure S.** Forest plot depicting the subgroup analysis PCOS vs. controls regarding fertilization rates (oocytes-based analysis), stratified by (a) stimulation with FSH, (b) priming with hCG.

**(a)**

**(b)**

**Figure T.** Forest plot depicting the subgroup analysis PCOS vs. PCO regarding fertilization rates (oocytes-based analysis), stratified by (a) stimulation with FSH, (b) priming with hCG.

**(a)**

**(b)**

**Figure U.** Forest plot depicting the comparison PCOS vs. non-PCOS regarding miscarriage rates (women-based analysis), stratified by (a) stimulation with FSH, (b) priming with hCG.

**(a)**

**(b)**

**Figure V.** Forest plot depicting the subgroup analysis regarding miscarriage rates (women-based analysis). (a) PCOS vs. healthy controls, (b) PCOS vs. PCO

**(a)**

**(b)**

**Figure W.** Secondary analysis within the non-PCOS group. Forest plot depicting the comparison PCO vs. controls regarding live birth rates. (a) cycles-based analysis; (b) women-based analysis.

**(a)**

**(b)**

**Figure X.** Secondary analysis within the non-PCOS group. Forest plot depicting the comparison PCO vs. controls regarding clinical pregnancy rates. (a) cycles-based analysis; (b) women-based analysis.

**(a)**

**(b)**

**Figure Y.** Secondary analysis within the non-PCOS group. Forest plot depicting the comparison PCO vs. controls regarding implantation rates (embryos-based analysis).

**Figure Z.** Secondary analysis within the non-PCOS group. Forest plot depicting the comparison PCO vs. controls regarding cancellation rates (cycles-based analysis).

**Figure AA.** Secondary analysis within the non-PCOS group. Forest plot depicting the comparison PCO vs. controls regarding maturation rates (oocytes-based analysis).

**Figure AB.** Secondary analysis within the non-PCOS group. Forest plot depicting the comparison PCO vs. controls regarding fertilization rates (oocytes-based analysis).

**Figure AC.** Secondary analysis within the non-PCOS group. Forest plot depicting the comparison PCO vs. controls regarding miscarriage rates (women-based analysis).

**Figure AD.** Forest plot depicting the comparison PCOS vs. non-PCOS regarding live birth rates, (a): cycles-based analysis, (b): women-based analysis, separately in study arms examining ICSI or IVF.

**(a)**

**(b)**

**Figure AE.** Forest plot depicting the comparison PCOS vs. non-PCOS regarding clinical pregnancy rates, (a) cycles-based analysis, (b) women-based analysis, separately in study arms examining ICSI or IVF.

**(a)**

**(b)**

**Figure AF.** Forest plot depicting the comparison PCOS vs. non-PCOS regarding (a) cancellation rates (cycles-based analysis), (b) maturation rates (oocytes-based analysis), separately in study arms examining ICSI or IVF.

**(a)**

**(b)**

**Figure AG.** Forest plot depicting the comparison PCOS vs. non-PCOS regarding (a) fertilization rates (oocytes-based analysis), (b) miscarriage rates (women-based analysis), separately in study arms examining ICSI or IVF.

**(a)**

**(b)**
