# Supplementary material for: In Vitro Maturation in Women with vs. without Polycystic Ovarian Syndrome: A Systematic Review and Meta-Analysis
Source: PLoS One. 2015 Aug 4;10(8):e0134696. doi: 10.1371/journal.pone.0134696 (PMC4524709; doi:10.1371/journal.pone.0134696)
Supplement: S2 File — Results of the meta-analyses addressing the comparison “PCO vs. controls” (analysis within non-PCOS women) regarding the examined outcomes. Bold cells denote statistically significant associations (Table B). Subgroup analyses by stimulation with FSH and priming with hCG, regarding the outcomes comprising four or more study arms; therefore no subgroup analyses are presented regarding implantation, as well as any of the “PCO vs. controls” comparisons. Bold cells denote statistically significant associations (Table C). Subgroup analyses by ICSI / IVF procedure; the study by Soderstrom-Anttila, 2005 was subdivided into two separate study arms for this analysis. Only outcomes comprising four or more published studies are presented; bold cells denote statistically significant associations (Table D). Evaluation of quality based on the Newcastle-Ottawa scale for all eleven included studies (Table E). (DOCX) [file pone.0134696.s002.docx]

**Table A:** Excluded studies and their references

| 1. Al-Sunaidi M | 2007 | Not comparative |
| --- | --- | --- |
| 1. Alcalay | 1995 | Not investigating research question of interest |
| 1. Beckers N | 1999 | Not comparative |
| 1. Benkhalifa M | 2009 | Not investigating research question of interest |
| 1. Bokal EV | 2005 | Not comparative |
| 1. Bos-Mikich A | 2011 | Not investigating research question of interest |
| 1. Cha and Chian | 1998 | Review |
| 1. Cha KY | 2005 | Not comparative |
| 1. Cha KY | 2006 | Review |
| 1. Cha KY | 2000 | Not investigating research question of interest |
| 1. Chian RC | 1999 | Not investigating research question of interest |
| 1. Chian RC | 2000 | Not investigating research question of interest |
| 1. Chian RC | 2004 | Case reports |
| 1. Child TJ | 2002 | Not investigating research question of interest |
| 1. Chung MK | 2000 | Not investigating research question of interest |
| 1. de Ziegler | 2012 | Not investigating research question of interest |
| 1. Ellenbogen A | 2011 | Not comparative |
| 1. Engmann L | 2008 | Not investigating research question of interest |
| 1. Farhi J | 2011 | Case series |
| 1. Filali M | 2008 | Not investigating research question of interest |
| 1. Fukuda AI | 2006 | Not comparative |
| 1. Ge HS | 2008 | Not investigating research question of interest |
| 1. Gremeau AS | 2012 | Not investigating research question of interest |
| 1. Griesinger G | 2006 | Review |
| 1. Grynberg | 2013 | Review |
| 1. Gulekli B | 2004 | Not investigating research question of interest |
| 1. Gulekli B | 2011 | Not investigating research question of interest |
| 1. Guzman | 2013 | Not investigating research question of interest |
| 1. Guzman L | 2013 | Not investigating research question of interest |
| 1. Harris SE | 2010 | IVM of surplus immature oocytes in other terms of research |
| 1. Harris SE | 2010 | Not investigating research question of interest |
| 1. Hashimoto S | 2007 | Not investigating research question of interest |
| 1. Holzer H | 2007 | Donor program |
| 1. Hourvitz A | 2010 | Not investigating research question of interest |
| 1. Hreinsson J | 2003 | Not investigating research question of interest |
| 1. Hyun CS | 2007 | Not investigating research question of interest |
| 1. Jayaprakasan K | 2012 | Not investigating research question of interest |
| 1. Jurema MW | 2006 | Review |
| 1. Kadoch IJ | 2007 | Not comparative |
| 1. Kdous M | 2009 | Not investigating research question of interest |
| 1. Kim | 2013 | Not investigating research question of interest |
| 1. Kolibianakis EM | 2007 | Not investigating research question of interest |
| 1. Lainas TG | 2007 | Not investigating research question of interest |
| 1. Le Du A | 2005 | Not comparative |
| 1. Li Y | 2005 | Not comparative |
| 1. Lin YH | 2003 | Not investigating research question of interest |
| 1. Lin YH | 2006 | Not investigating research question of interest |
| 1. Liu JY | 2003 | Not enough data to answer the research question of interest |
| 1. Liu S | 2010 | Not comparative |
| 1. Lornage J | 2006 | Not comparative |
| 1. Marci R | 2001 | Not investigating research question of interest |
| 1. Mikkelsen AL | 2005 | Not comparative, review |
| 1. Mikkelsen AL | 2001 | Not investigating research question of interest |
| 1. Mikkelsen AL | 2003 | Not investigating research question of interest |
| 1. Moosova M | 2011 | Not investigating research question of interest |
| 1. Ortega-Hrepich | 2013 | Not investigating research question of interest |
| 1. Orvieto R | 2009 | Not investigating research question of interest |
| 1. Ouandaogo | 2012 | Not investigating research question of interest |
| 1. Pagidas K | 2010 | Not investigating research question of interest |
| 1. Papanikolaou EG | 2005 | Review |
| 1. Patel SS | 2008 | Review |
| 1. Qian Y | 2005 | Not investigating research question of interest |
| 1. Rao GD | 2005 | Not investigating research question of interest |
| 1. Roesner S | 2012 | Not enough data to answer the research question of interest |
| 1. Sahu B | 2008 | Not investigating research question of interest |
| 1. Shalom-Paz E | 2012 | Not investigating research question of interest |
| 1. Shulman A | 1997 | Review |
| 1. Siristatidis C | 2009 | Review |
| 1. Siristatidis C | 2013 | Review |
| 1. Son WY | 2005 | Not investigating research question of interest |
| 1. Son WY | 2007 | Not investigating research question of interest |
| 1. Son WY | 2008 | Not investigating research question of interest |
| 1. Son WY | 2008 | Not investigating research question of interest |
| 1. Son WY | 2008 | Not investigating research question of interest |
| 1. Son WY | 2009 | Not investigating research question of interest |
| 1. Son WY | 2010 | Review |
| 1. Son WY | 2011 | Not investigating research question of interest |
| 1. Suikkari AM | 2007 | Review |
| 1. van Wely M | 2005 | Not investigating research question of interest |
| 1. Vieira RC | 2008 | Results reported in the included study by Vieira, 2011 |
| 1. Vieira RC | 2008 | Not investigating research question of interest |
| 1. Vlaisavljevic V | 2009 | Not investigating research question of interest |
| 1. Wachs DS | 2006 | Not investigating research question of interest |
| 1. Walls M | 2012 | Not investigating research question of interest |
| 1. Wei Z | 2008 | Not investigating research question of interest |
| 1. Wiser | 2013 | Not investigating research question of interest |
| 1. Xu YP | 2012 | Not investigating research question of interest |
| 1. Yang SH | 2005 | Not investigating research question of interest |
| 1. Yu R | 2012 | Not investigating research question of interest |
| 1. Zhao JZ | 2009 | Not comparative |
| 1. Zheng X | 2012 | Not investigating research question of interest |

**References**

1. Al-Sunaidi M, Tulandi T, Holzer H, Sylvestre C, Chian RC, Tan SL. Repeated pregnancies and live births after in vitro maturation treatment. Fertil Steril 2007;87(5):1212.e9-12.
2. Alcalay M, Bider D, Lipitz S, Mashiach S, Levran D, Dor J. Polycystic ovarian syndrome: pregnancy outcome following in vitro fertilization-embryo transfer. Gynecol Endocrinol 1995;9(2):119-23.
3. Beckers N, Pieters M, Ramos L, Zeilmaker G, Fauser B, Braat D. Retrieval, maturation, and fertilization of immature oocytes obtained from unstimulated patients with polycystic ovary syndrome. J Assist Reprod Genet 1999:16; 81-6.
4. Benkhalifa M, Demirol A, Ménézo Y, Balashova E, Abduljalil AK, Abbas S, et al. Natural cycle IVF and oocyte in-vitro maturation in polycystic ovary syndrome: a collaborative prospective study. Reprod Biomed Online 2009;18(1):29-36.
5. Bokal EV, Vrtovec HM, Virant Klun I, Verdenik I. Prolonged HCG action affects angiogenic substances and improves follicular maturation, oocyte quality and fertilization competence in patients with polycystic ovarian syndrome. Hum Reprod 2005;20(6):1562-8.
6. Bos-Mikich A, Ferreira M, Höher M, Frantz G, Oliveira N, Dutra CG, et al. Fertilization outcome, embryo development and birth after unstimulated IVM. J Assist Reprod Genet 2011;28:107-10.
7. Cha KY, Chian RC. Maturation in vitro of immature human oocytes for clinical use. Hum Reprod Update 1998;4:103–20.
8. Cha KY, Chung HM, Lee DR, Kwon H, Chung MK, Park LS, et al. Obstetric outcome of patients with polycystic ovary syndrome treated by in vitro maturation and in vitro fertilization-embryo transfer. Fertil Steril 2005;83:1461-5.
9. Cha KY, Lee DR, Cho JH, Yoon TK. In vitro maturation of immature oocytes and IVF/ICSI in PCOS patients. J Indian Med Assoc 2006;104(8):446, 448, 473.
10. Cha KY, Han SY, Chung HM, Choi DH. Pregnancies and deliveries after in vitro maturation culture followed by in vitro fertilization and embryo transfer without stimulation in women with polycystic ovary syndrome. Fertil Steril 2000;73:978-83.
11. Chian RC, Gulekli B, Buckett WM, Tan SL. Priming with human chorionic gonadotropin before retrieval of immature oocytes in women with infertility due to the polycystic ovary syndrome. NEJM 1999;341:1624-6.
12. Chian RC, Buckett WM, Tulandi T, Tan SL. Prospective randomized study of human chorionic gonadotrophin priming before immature oocyte retrieval from unstimulated women with polycystic ovarian syndrome. Hum Reprod 2000;15:165-70.
13. Chian RC, Buckett WM, Jalil A, Son WY, Sylvestre C, Rao D, et al. Natural cycle in vitro fertilization combined with in vitro maturation of immature oocytes is a potential approach in infertility treatment. Fertil Steril 2004;82: 1675-8.
14. Child TJ, Phillips SJ, Abdul-Jalil AK, Gulakli B, Tan SL. A comparison of in vitro maturation and in vitro fertilization for women with polycystic ovaries. Obstet Gynecol 2002;100:665-70.
15. Chung MK, Chung HM, Lee WS, Han SY, Yoon TK, Cha KY. Applicability of a hCG priming of unstimulated PCOS patients for improving IVM/IVF-ET outcome. Fertil Steril 2000;74:32-3.
16. de Ziegler D, Streuli I, Gayet V, Frydman N, Bajouh O, Chapron C. Retrieving oocytes from small non-stimulated follicles in polycystic ovary syndrome (PCOS): in vitro maturation (IVM) is not indicated in the new GnRH antagonist era. Fertil Steril 2012;98(2):290-3.
17. Ellenbogen A, Atamny R, Fainaru O, Meidan E, Rotfarb N, Michaeli M. In vitro maturation of oocytes: a novel method of treatment of patients with polycystic ovarian syndrome undergoing in vitro fertilization. Harefuah 2011;150(11):833-6, 876.
18. Engmann L, DiLuigi A, Schmidt D, Nulsen J, Maier D, Benadiva C. The use of gonadotropin-releasing hormone (GnRH) agonist to induce oocyte maturation after cotreatment with GnRH antagonist in high-risk patients undergoing in vitro fertilization prevents the risk of ovarian hyperstimulation syndrome: a prospective randomized controlled study. Fertil Steril 2008;89(1):84-91.
19. Farhi J, Sapir O, Maman M, Fisch B, Ben-Haroush A. Novel protocol for scheduling oocyte retrieval in IVM cycles in PCOS patients: a case series. Reprod Biomed Online 2011;23(6):765-8.
20. Filali M, Hesters L, Fanchin R, Tachdjian G, Frydman R, Frydman N. Retrospective comparison of two media for invitro maturation of oocytes. Reprod Biomed Online 2008;16:250-6.
21. Fukuda AI, Sato M, Sugihara K, Nagata F, Nakaoka Y, Morimoto Y. In vitro maturation, in vitro fertilisation and embryo transfer (IVM-IVF) combined with low dose FSH over metformin pretreatment and frozen-thawed cycles should be a routine ART option for polycystic ovarian syndrome (PCOS) patients. Fertil Steril 2006;86:129.
22. Ge HS, Huang XF, Zhang W, Zhao JZ, Lin JJ, Zhou WZ. Exposure to human chorionic gonadotropin during in vitro maturation does not improve the maturation rate and developmental potential of immature oocytes from patients with polycystic ovary syndrome. Fertil Steril 2008;89:98-103.
23. Gremeau AS, Andreadis N, Fatum M, Craig J, Turner K, McVeigh E, et al. In vitro maturation or in vitro fertilization for women with polycystic ovaries? A case-control study of 194 treatment cycles. Fertil Steril 2012;98(2):355-60.
24. Griesinger G, Diedrich K, Tarlatzis BC, Kolibianakis EM. GnRH-antagonists in ovarian stimulation for IVF in patients with poor response to gonadotrophins, polycystic ovary syndrome, and risk of ovarian hyperstimulation: a meta-analysis. Reprod Biomed Online. 2006;13(5):628-38.
25. Grynberg M, El Hachem H, de Bantel A, Benard J, le Parco S, Fanchin R. In vitro maturation of oocytes: uncommon indications. Fertil Steril 2013;99(5):1182-8.
26. Gulekli B, Buckett WM, Chian RC, Child TJ, Abdul-Jalil AK, Tan SL. Randomized, controlled trial of priming with 10,000 IU versus 20,000 IU of human chorionic gonadotropin in women with polycystic ovary syndrome who are undergoing in vitro maturation. Fertil Steril 2004;82:1458-9.
27. Gulekli B, Kovali M, Aydiner F, Dogan S, Dogan SS. IVM is an alternative for patients with PCO after failed conventional IVF attempt. J Assist Reprod Genet 2011;28(6):495-9.
28. Guzman L, Ortega-Hrepich C, Polyzos NP, Anckaert E, Verheyen G, Coucke W, et al. A prediction model to select PCOS patients suitable for IVM treatment based on anti-Mullerian hormone and antral follicle count. Hum Reprod 2013;28(5):1261-6.
29. Guzman L, Adriaenssens T, Ortega-Hrepich C, Albuz FK, Mateizel I, Devroey P, et al. Human antral follicles <6 mm: a comparison between in vivo maturation and in vitro maturation in non-hCG primed cycles using cumulus cell gene expression. Mol Hum Reprod 2013;19(1):7-16.
30. Harris SE, Maruthini D, Tang T, Balen AH, Picton HM. Metabolism and karyotype analysis of oocytes from patients with polycystic ovary syndrome. Hum Reprod 2010;25:2305-15.
31. Hashimoto S, Fukuda A, Murata Y, Kikkawa M, Oku H, Kanaya H, et al. Effect of aspiration vacuum on the developmental competence of immature human oocytes retrieved using a 20-gauge needle. Reprod Biomed Online 2007;14(4):444-9.
32. Holzer H, Scharf E, Chian RC, Demirtas E, Buckett W, Tan SL. In vitro maturation of oocytes collected from unstimulated ovaries for oocyte donation. Fertil Steril 2007;88:62-7.
33. Hourvitz A, Maman E, Brengauz M, Machtinger R, Dor J. In vitro maturation for patients with repeated in vitro fertilization failure due to "oocyte maturation abnormalities". Fertil Steril 2010;94(2):496-501.
34. Hreinsson J, Rosenlund B, Friden B, Levkov L, Ek I, Suikkari AM, et al. Recombinant LH is equally effective as recombinant HCG in promoting oocyte maturation in a clinical in vitro maturation program: a randomised study. Hum Reprod 2003;18:2131-6.
35. Hyun CS, Cha JH, Son WY, Yoon SH, Kim KA, Lim JH. Optimal ICSI timing after the first polar body extrusion in in vitro matured human oocytes. Hum Reprod. 2007;22(7):1991-5.
36. Jayaprakasan K, Chan Y, Islam R, Haoula Z, Hopkisson J, Coomarasamy A, Raine-Fenning N. Prediction of in vitro fertilization outcome at different antral follicle count thresholds in a prospective cohort of 1,012 women. Fertil Steril. 2012;98(3):657-63.
37. Kadoch IJ, Fanchin R, Frydman N, Le Du A, Frydman R. Controlled natural cycle IVF: a novel approach for a dominant follicle during an in-vitro maturation cycle. Reprod Biomed Online 2007;14(5):598-601.
38. Kdous M, Chaker A, Bouyahia M, Zhioua F, Zhioua A. Increased risk of early pregnancy loss and lower live birth rate with GNRH antagonist vs. long GNRH agonist protocol in PCOS women undergoing controlled ovarian hyperstimulation. Tunis Med 2009;87(12):834-42.
39. Kim MK, Park EA, Kim HJ, Choi WY, Cho JH, Lee WS, et al. Does supplementation of in-vitro culture medium with melatonin improve IVF outcome in PCOS? Reprod Biomed Online 2013;26(1):22-9.
40. Kolibianakis EM, Papanikolaou EG, Tournaye H, Camus M, Van Steirteghem AC, Devroey P. Triggering final oocyte maturation using different doses of human chorionic gonadotropin: a randomized pilot study in patients with polycystic ovary syndrome treated with gonadotropin-releasing hormone antagonists and recombinant follicle-stimulating hormone. Fertil Steril 2007;88(5):1382-8.
41. Lainas TG, Petsas GK, Zorzovilis IZ, Iliadis GS, Lainas GT, Cazlaris HE, et al. Initiation of GnRH antagonist on Day 1 of stimulation as compared to the long agonist protocol in PCOS patients. A randomized controlled trial: effect on hormonal levels and follicular development. Hum Reprod 2007;22(6):1540-6.
42. Le Du A, Kadoch IJ, Bourcigaux N, Doumerc S, Bourrier MC, Chevalier N, et al. In vitro oocyte maturation for the treatment of infertility associated with polycystic ovarian syndrome: the French experience. Hum Reprod 2005;20:420-4.
43. Li Y, Jiang JJ, Ma SY, Li M, Hu JM, Zhao LX, Chen ZJ. In vitro maturation and fertilization of oocytes from unstimulated cycles in women with infertility due to polycystic ovary syndrome. Zhonghua Fu Chan Ke Za Zhi 2005;40(6):388-91.
44. Lin YH, Hwang JL, Huang LW, Mu SC, Seow KM, Chung J, et al. Combination of FSH priming and hCG priming for in-vitro maturation of human oocytes. Hum Reprod 2003;18:1632-6.
45. Liu JY, Qian Y, Mao YD, Ding W, Ynag NM. In vitro maturation, fertilization and embryo transfer of human immature oocyte. Chin J Obstet Gynecol 2003;38:230-2.
46. Liu S, Jiang JJ, Feng HL, Ma SY, Li M, Li Y. Evaluation of the immature human oocytes from unstimulated cycles in polycystic ovary syndrome patients using a novel scoring system. Fertil Steril 2010;93:2202-9.
47. Lornage J. In vitro maturation: the French experience. J Gynecol Obstet Biol Reprod (Paris). 2006;35(5 Pt 2):2S3-2S7.
48. Marci R, Senn A, Dessole S, Chanson A, Loumaye E, De Grandi P, Germond M. A low-dose stimulation protocol using highly purified follicle-stimulating hormone can lead to high pregnancy rates in in vitro fertilization patients with polycystic ovaries who are at risk of a high ovarian response to gonadotropins. Fertil Steril 2001;75(6):1131-5.
49. Mikkelsen AL. Strategies in human in-vitro maturation and their clinical outcome. Reprod Biomed Online 2005;10(5):593-9.
50. Mikkelsen AL, Lindenberg S. Benefit of FSH priming of women with PCOS to the in vitro maturation procedure and the outcome: A randomized prospective study. Reproduction 2001;122:587-92.
51. Mikkelsen AL, Host E, Blaabjerg J, Lindenberg S. Time interval between FSH priming and aspiration of immature human oocytes for in vitro maturation: a prospective randomised study. Reprod Biomed Online 2003;6:416-20.
52. Moosová M, Moos J, Filová V, Pavelková J, Jarosová R, Rezábek K. A relation between the polycystic ovary syndrome and the status of ovarian hyperstimulation syndrome in IVF patients after controlled hyperstimulation. Ceska Gynekol. 2011;76(2):123-7.
53. Ortega-Hrepich C, Stoop D, Guzman L, Van Landuyt L, Tournaye H, Smitz J, et al. A "freeze-all" embryo strategy after¬†in¬†vitro maturation: a novel approach in women with polycystic ovary syndrome? Fertil Steril 2013;100(4):1002-1007.e1.
54. Orvieto R, Meltcer S, Homburg R, Nahum R, Rabinson J, Ashkenazi J. What is the preferred GnRH analogue for polycystic ovary syndrome patients undergoing controlled ovarian hyperstimulation for in vitro fertilization? Fertil Steril. 2009;91(4 Suppl):1466-8.
55. Ouandaogo ZG, Frydman N, Hesters L, Assou S, Haouzi D, Dechaud H, et al. Differences in transcriptomic profiles of human cumulus cells isolated from oocytes at GV, MI and MII stages after in vivo and in vitro oocyte maturation. Hum Reprod. 2012;27(8):2438-47.
56. Pagidas K, Carson SA, McGovern PG, Barnhart HX, Myers ER, Legro RS, et al. Intercourse compliance, ovulation, and treatment success in the National Institute of Child Health and Human Development-Reproductive Medicine Network's Pregnancy in Polycystic Ovary Syndrome (PPCOS) Trial. Fertil Steril. 2010;94(4):1444-6.
57. Papanikolaou EG, Platteau P, Albano C, Nogueira D, Cortvrindt R, Devroey P, Smitz J. Immature oocyte in-vitro maturation: clinical aspects. Reprod Biomed Online 2005;10(5):587-92.
58. Patel SS, Carr BR. Oocyte quality in adult polycystic ovary syndrome. Semin Reprod Med 2008;26(2):196-203.
59. Qian Y, Feng T, Chen J, Cai LB, Liu JY, Mao YD, Ding W, Sha JH. Fertilization of in vitro matured human oocytes by intracytoplasmic sperm injection (ICSI) using ejaculated and testicular spermatozoa. Asian J Androl 2005;7(1):39-43.
60. Rao GD, Tan SL. In vitro maturation of oocytes. Semin Reprod Med 2005;23(3):242-7.
61. Roesner S, Von Wolff M, Eberhardt I, Beuter-Winkler P, Toth B, Strowitzki T. In vitro maturation: a five-year experience. Acta Obstet Gynecol Scand 2012;91(1):22-7.
62. Sahu B, Ozturk O, Ranierri M, Serhal P. Comparison of oocyte quality and intracytoplasmic sperm injection outcome in women with isolated polycystic ovaries or polycystic ovarian syndrome. Arch Gynecol Obstet 2008;277(3):239-44.
63. Shalom-Paz E, Holzer H, Son W, Levin I, Tan SL, Almog B. PCOS patients can benefit from in vitro maturation (IVM) of oocytes. Eur J Obstet Gynecol Reprod Biol 2012;165(1):53-6.
64. Shulman A, Dor J. In vitro fertilization treatment in patients with polycystic ovaries. J Assist Reprod Genet 1997;14(1):7-10.
65. Siristatidis CS, Vrachnis N, Creatsa M, Maheshwari A, Bhattacharya S. In vitro maturation in subfertile women with polycystic ovarian syndrome undergoing assisted reproduction. Cochrane Database Syst Rev 2013;10:CD006606.
66. Siristatidis CS, Maheshwari A, Bhattacharya S. In vitro maturation in sub fertile women with polycystic ovarian syndrome undergoing assisted reproduction. Cochrane Database Syst Rev 2009;(1):CD006606.
67. Son WY, Chung JT, Herrero B, Dean N, Demirtas E, Holzer H, et al. Selection of the optimal day for oocyte retrieval based on the diameter of the dominant follicle in hCG-primed in vitro maturation cycles. Hum Reprod. 2008;23(12):2680-5.
68. Son WY, Chung JT, Dahan M, Reinblatt S, Tan SL, Holzer H. Comparison of fertilization and embryonic development in sibling in vivo matured oocytes retrieved from different sizes follicles from in vitro maturation cycles. J Assist Reprod Genet 2011;28(6):539-44.
69. Son WY, Chung JT, Gidoni Y, Holzer H, Levin D, Chian RC, et al. Comparison of survival rate of cleavage stage embryos produced from in vitro maturation cycles after slow freezing and after vitrification. Fertil Steril 2009;92(3):956-8.
70. Son WY, Chung JT, Chian RC, Herrero B, Demirtas E, Elizur S, et al. A 38 h interval between hCG priming and oocyte retrieval increases in vivo and in vitro oocyte maturation rate in programmed IVM cycles. Hum Reprod. 2008;23(9):2010-6.
71. Son WY, Chung JT, Demirtas E, Holzer H, Sylvestre C, Buckett W, et al. Comparison of in-vitro maturation cycles with and without in-vivo matured oocytes retrieved. RBM Online 2008;17.n
72. Son WY, Lee SY, Yoon SH, Lim JH. Pregnancies and deliveries after transfer of human blastocysts derived from in vitro matured oocytes in in vitro maturation cycles. Fertil Steril 2007;87:1491-3.
73. Son WY, Tan SL. Laboratory and embryological aspects of hCG-primed in vitro maturation cycles for patients with polycystic ovaries. Human Reprod Update 2010;16:675–89.
74. Son WY, Lee SY, Lim JH. Fertilization, cleavage and blastocyst development according to the maturation timing of oocytes in in vitro maturation cycles. Hum Reprod 2005;20:3204-7.
75. Suikkari AM, Söderström-Anttila V. In-vitro maturation of eggs: is it really useful? Best Pract Res Clin Obstet Gynaecol 2007;21(1):145-55.
76. van Wely M, Bayram N, van der Veen F, Bossuyt PM. Predicting ongoing pregnancy following ovulation induction with recombinant FSH in women with polycystic ovary syndrome. Hum Reprod. 2005;20(7):1827-32.
77. Vieira RC, Barcelos ID, Ferreira EM, de Araújo MC, dos Reis RM, Ferriani RA, et al. Evaluation of meiotic abnormalities of oocytes from polycystic ovary syndrome patients submitted to ovarian stimulation. Rev Bras Ginecol Obstet 2008;30:241-7.
78. Vlaisavljević V, Kovac V, Sajko MC. Impact of insulin resistance on the developmental potential of immature oocytes retrieved from human chorionic gonadotropin-primed women with polycystic ovary syndrome undergoing in vitro maturation. Fertil Steril 2009;91(3):957-9.
79. Wachs DS, Coffler MS, Malcom PJ, Chang RJ. Comparison of follicle-stimulating-hormone-stimulated dimeric inhibin and estradiol responses as indicators of granulosa cell function in polycystic ovary syndrome and normal women. J Clin Endocrinol Metab 2006;91(8):2920-5.
80. [Walls M](http://www.ncbi.nlm.nih.gov/pubmed?term=Walls%20M%5BAuthor%5D&cauthor=true&cauthor_uid=23063820), [Junk S](http://www.ncbi.nlm.nih.gov/pubmed?term=Junk%20S%5BAuthor%5D&cauthor=true&cauthor_uid=23063820), [Ryan JP](http://www.ncbi.nlm.nih.gov/pubmed?term=Ryan%20JP%5BAuthor%5D&cauthor=true&cauthor_uid=23063820), [Hart R](http://www.ncbi.nlm.nih.gov/pubmed?term=Hart%20R%5BAuthor%5D&cauthor=true&cauthor_uid=23063820). IVF versus ICSI for the fertilization of in-vitro matured human oocytes. Reprod Biomed Online 2012;25(6):603-7.
81. Wei Z, Cao Y, Cong L, Zhou P, Zhang Z, Li J. Effect of metformin pretreatment on pregnancy outcome of in vitro matured oocytes retrieved from women with polycystic ovary syndrome. Fertil Steril. 2008;90(4):1149-54.
82. Wiser A, Shehata F, Holzer H, Hyman JH, Shalom-Paz E, Son WY, et al. Effect of high LH/FSH ratio on women with polycystic ovary syndrome undergoing in vitro maturation treatment. J Reprod Med 2013;58(5-6):219-23.
83. Xu YP, Xiang HF, Zou WW, Li ZL, Zhang ZG, Zhou P, et al. Clinical application of in vitro maturation of human immature oocytes for infertile women with polycystic ovary syndrome. Zhonghua Fu Chan Ke Za Zhi 2012;47(1):14-8.
84. Yang SH, Son WY, Yoon SH, Ko Y, Lim JH. Correlation between in vitro maturation and expression of LH receptor in cumulus cells of the oocytes collected from PCOS patients in HCG-primed IVM cycles. Hum Reprod 2005;20(8):2097-103.
85. Yu R, Lin J, Zhao JZ, Wang PY, Xiao SQ, Zhang W. [Study on clinical effect on infertility women with polycystic ovary syndrome treated by in vitro maturation and in vitro fertilization-embryo transfer]. Zhonghua Fu Chan Ke Za Zhi 2012;47(4):250-4.
86. Zhao JZ, Chen X, Wang PY, Zhou W, Lin JJ, Zhang W, et al. Outcome of pregnancy in women with polycystic ovary syndrome treated by in vitro maturation of immature oocytes. Chin J Obstet Gynecol 2009;44:409-12.
87. Zheng X, Wang L, Zhen X, Lian Y, Liu P, Qiao J. Effect of hCG priming on embryonic development of immature oocytes collected from unstimulated women with polycystic ovarian syndrome. Reprod Biol Endocrinol 2012;10:40.
88. Harris SE, Maruthini D, Tang T, Balen AH, Picton HM. Metabolism and karyotype analysis of oocytes from patients with polycystic ovary syndrome. Hum Reprod 2010;25:2305-15.
89. Vieira RC, Barcelos ID, Ferreira EM, de Araújo MC, dos Reis RM, Ferriani RA, et al. Evaluation of meiotic abnormalities of oocytes from polycystic ovary syndrome patients submitted to ovarian stimulation. Rev Bras Ginecol Obstet 2008;30:241-7.
90. Lin YH and Hwang JL. In vitro maturation of human oocytes. Taiwanese J Obstet Gynecol 2006;45:95-9.
91. Jurema MW and Nogueira D. In vitro maturation of human oocytes for assisted reproduction. Fertil Steril 2006;86:1277-91.

**Table B.** Results of the meta-analyses addressing the comparison “PCO vs. controls” (analysis within non-PCOS women) regarding the examined outcomes. Bold cells denote statistically significant associations.

|  | PCO vs. controls | | |
| --- | --- | --- | --- |
|  | n^§^ | OR (95%CI) | Heterogeneity  I^2^, p |
| Live birth  (cycles-based analysis) | 2 | 2.50 (0.16-39.09) | 77.9%, 0.033 |
| Live birth  (women-based analysis) | 2 | 2.62 (0.17-41.46) | 78.0%, 0.033 |
| Clinical pregnancy  (cycles-based analysis) | 2 | 1.93 (0.12-30.17) | 84.5%, 0.011 |
| Clinical pregnancy  (women-based analysis) | 2 | 2.00 (0.12-33.63) | 85.2%, 0.009 |
| Implantation  (embryos-based analysis) | 2 | 1.57 (0.11-23.35) | 84.8%, 0.010 |
| Cancellation  (cycles-based analysis) | 2 | 0.57 (0.08-4.30) | 66.7%, 0.083 |
| Maturation  (oocytes-based analysis) | 2 | 0.88 (0.70-1.10) | 0.0%, 0.986 |
| Fertilization  (oocytes-based analysis) | 2 | 0.99 (0.64-1.52) | 59.9%, 0.114 |
| Miscarriage  (women-based analysis) | 2 | 0.44 (0.55-3.90) | 0.0%, 0.805 |

^§^number of study arms

**Table C.** Subgroup analyses by stimulation with FSH and priming with hCG, regarding the outcomes comprising four or more study arms; therefore no subgroup analyses are presented regarding implantation, as well as any of the “PCO vs. controls” comparisons. Bold cells denote statistically significant associations.

|  | PCOS vs. non-PCOS (PCO/controls) | | | Subgroup analysis:  PCOS vs. controls | | | Subgroup analysis:  PCOS vs. PCO | | |
| --- | --- | --- | --- | --- | --- | --- | --- | --- | --- |
|  | n^§^ | OR (95%CI) | Heterogeneity  I^2^, p | n^§^ | OR (95%CI) | Heterogeneity  I^2^, p | n^§^ | OR (95%CI) | Heterogeneity  I^2^, p |
| **Analysis stratified by stimulation with FSH** |  |  |  |  |  |  |  |  |  |
| Live birth  (cycles-based analysis) | 4 | 1.56 (0.90-2.72) | 0.0%, 0.766 |  | Not applicable |  | 4 | 1.09 (0.57-2.10) | 0.0%, 0.643 |
| *Stimulation with FSH* | 2 | 1.00 (0.36-2.77) | 0.0%, 0.927 |  |  |  | 2 | 1.00 (0.36-2.77) | 0.0%, 0.927 |
| *No stimulation with FSH* | 2 | 1.89 (0.97-3.66) | 0.0%, 0.764 |  |  |  | 2 | 1.31 (0.40-4.28) | 38.1%, 0.204 |
|  |  |  |  |  |  |  |  |  |  |
| Live birth  (women-based analysis) | 4 | 1.74 (0.99-3.04) | 0.0%,0.693 |  | Not applicable |  | 4 | 1.18 (0.61-2.29) | 0.0%, 0.604 |
| *Stimulation with FSH* | 2 | 1.07 (0.39-2.97) | 0.0%, 0.778 |  |  |  | 2 | 1.07 (0.39-2.97) | 0.0%, 0.778 |
| *No stimulation with FSH* | 2 | **2.14 (1.10-4.18)** | 0.0%, 0.710 |  |  |  | 2 | 1.44 (0.42-4.96) | 41.4%, 0.191 |
|  |  |  |  |  |  |  |  |  |  |
| Clinical pregnancy  (cycles-based analysis) | 7 | **2.23 (1.45-3.43)** | 0.0%, 0.722 | 4 | **3.09 (1.46-6.53)** | 36.8%, 0.191 | 5 | 1.60 (0.89-2.88) | 0.0%, 0.552 |
| *Stimulation with FSH* | 3 | 1.16 (0.52-2.58) | 0.0%, 0.988 | 1 | 1.26 (0.35-4.56) | NC | 2 | 1.11 (0.40-3.06) | 0.0%, 0.991 |
| *No stimulation with FSH* | 4 | **2.92 (1.75-4.86)** | 0.0%, 0.999 | 3 | **3.89 (1.82-8.29)** | 22.9%, 0.273 | 3 | 2.08 (0.88-4.93) | 12.4%, 0.319 |
|  |  |  |  |  |  |  |  |  |  |
| Clinical pregnancy  (women-based analysis) | 7 | **2.37 (1.53-3.68)** | 0.0%, 0.666 | 4 | **3.29 (1.42-7.62)** | 46.8%, 0.131 | 5 | 1.75 (0.96-3.20) | 0.0%, 0.586 |
| *Stimulation with FSH* | 3 | 1.22 (0.55-2.70) | 0.0%, 0.930 | 1 | 1.26 (0.35-4.56) | NC | 2 | 1.19 (0.43-3.28) | 0.0%, 0.709 |
| *No stimulation with FSH* | 4 | **3.17 (1.87-5.37)** | 0.0%, 0.995 | 3 | **4.35 (1.77-10.67)** | 39.0%, 0.194 | 3 | **2.16 (1.02-4.57)** | 0.0%, 0.399 |
|  |  |  |  |  |  |  |  |  |  |
| Cancellation  (cycles-based analysis) | 5 | **0.18 (0.06-0.47)** | 0.0%,0.895 |  | Not applicable |  | 4 | **0.25 (0.07-0.92)** | 0.0%, 0.783 |
| *Stimulation with FSH* | 2 | 0.20 (0.01-3.27) | 0.0%, 0.600 |  |  |  | 2 | 0.20 (0.01-3.27) | 0.0%, 0.600 |
| *No stimulation with FSH* | 3 | **0.17 (0.06-0.50)** | 0.0%, 0.665 |  |  |  | 2 | 0.27 (0.06-1.17) | 0.0%,0.382 |
|  |  |  |  |  |  |  |  |  |  |
| Maturation  (oocytes-based analysis) | 8 | 0.87 (0.70-1.08) | 63.7%, 0.007 | 5 | **0.74 (0.59-0.93)** | 52.4%, 0.078 | 5 | 1.03 (0.88-1.21) | 0.0%, 0.734 |
| *Stimulation with FSH* | 4 | 0.98 (0.63-1.52) | 75.0%, 0.007 | 2 | 0.81 (0.39-1.67) | 65.7%, 0.088 | 2 | 1.14 (0.84-1.56) | 0.0%, 0.390 |
| *No stimulation with FSH* | 4 | 0.84 (0.65-1.08) | 55.1%, 0.083 | 3 | 0.76 (0.57-1.01) | 48.2%, 0.145 | 3 | 0.99 (0.88-1.20) | 0.0%, 0.708 |
|  |  |  |  |  |  |  |  |  |  |
| Fertilization  (oocytes-based analysis) | 8 | 0.78 (0.60-1.03) | 71.3%, 0.001 | 5 | 0.76 (0.55-1.04) | 72.9%, 0.005 | 5 | 0.88 (0.54-1.43) | 79.9%, 0.001 |
| *Stimulation with FSH* | 4 | **0.74 (0.61-0.89)** | 0.0%, 0.451 | 2 | 0.74 (0.52-1.05) | 42.2%, 0.188 | 2 | 0.87 (0.58-1.32) | 0.0%, 0.852 |
| *No stimulation with FSH* | 4 | 0.76 (0.44-1.30) | 86.2%, <0.001 | 3 | 0.73 (0.41-1.30) | 83.3%, 0.003 | 3 | 0.88 (0.40-1.93) | 89.4%,<0.001 |
|  |  |  |  |  |  |  |  |  |  |
| Miscarriage  (women-based analysis) | 4 | 1.51 (0.72-3.17) | 0.0%, 0.795 |  | Not applicable |  |  | Not applicable |  |
| *Stimulation with FSH* | 1 | 1.19 (0.04-32.08) | NC |  |  |  |  |  |  |
| *No stimulation with FSH* | 3 | 1.53 (0.72-3.27) | 0.0%, 0.606 |  |  |  |  |  |  |
|  |  |  |  |  |  |  |  |  |  |
|  |  |  |  |  |  |  |  |  |  |
| **Analysis stratified by priming with hCG** |  |  |  |  |  |  |  |  |  |
| Live birth  (cycles-based analysis) | 4 | 1.56 (0.90-2.72) | 0.0%, 0.766 |  | Not applicable |  | 4 | 1.09 (0.57-2.10) | 0.0%, 0.643 |
| *Priming with hCG* | 2 | 1.89 (0.97-3.66) | 0.0%, 0.764 |  |  |  | 2 | 1.31 (0.40-4.28) | 38.1%, 0.204 |
| *No priming with hCG* | 2 | 1.00 (0.36-2.77) | 0.0%, 0.927 |  |  |  | 2 | 1.00 (0.36-2.77) | 0.0%, 0.927 |
|  |  |  |  |  |  |  |  |  |  |
| Live birth  (women-based analysis) | 4 | 1.74 (0.99-3.04) | 0.0%,0.693 |  | Not applicable |  | 4 | 1.18 (0.61-2.29) | 0.0%, 0.604 |
| *Priming with hCG* | 2 | **2.14 (1.10-4.18)** | 0.0%, 0.710 |  |  |  | 2 | 1.44 (0.42-4.96) | 41.4%, 0.191 |
| *No priming with hCG* | 2 | 1.07 (0.39-2.97) | 0.0%, 0.778 |  |  |  | 2 | 1.07 (0.39-2.97) | 0.0%, 0.778 |
|  |  |  |  |  |  |  |  |  |  |
| Clinical pregnancy  (cycles-based analysis) | 7 | **2.23 (1.45-3.43)** | 0.0%, 0.722 | 4 | **3.09 (1.46-6.53)** | 36.8%, 0.191 | 5 | 1.60 (0.89-2.88) | 0.0%, 0.552 |
| *Priming with hCG* | 2 | **2.88 (1.64-5.07)** | 0.0%, 0.943 | 2 | **4.84 (1.27-18.45)** | 60.3%, 0.112 | 2 | 2.39 (0.63-9.04) | 55.5%, 0.134 |
| *No priming with hCG* | 5 | 1.57 (0.81-3.05) | 0.0%, 0.774 | 2 | 1.99 (0.80-4.95) | 0.0%, 0.323 | 3 | 1.19 (0.45-3.15) | 0.0%, 0.888 |
|  |  |  |  |  |  |  |  |  |  |
| Clinical pregnancy  (women-based analysis) | 7 | **2.37 (1.53-3.68)** | 0.0%, 0.666 | 4 | **3.29 (1.42-7.62)** | 46.8%, 0.131 | 5 | 1.75 (0.96-3.20) | 0.0%, 0.586 |
| *Priming with hCG* | 2 | **3.15 (1.77-5.61)** | 0.0%, 0.828 | 2 | **5.43 (1.16-25.39)** | 69.1%, 0.072 | 2 | 2.52 (0.76-8.31) | 45.2%, 0.177 |
| *No priming with hCG* | 5 | 1.60 (0.81-3.15) | 0.0%, 0.770 | 2 | 1.99 (0.75-5.27) | 5.3%, 0.304 | 3 | 1.27 (0.48-3.36) | 0.0%, 0.844 |
|  |  |  |  |  |  |  |  |  |  |
| Cancellation  (cycles-based analysis) | 5 | **0.18 (0.06-0.47)** | 0.0%,0.895 |  | Not applicable |  | 4 | **0.25 (0.07-0.92)** | 0.0%, 0.783 |
| *Priming with hCG* | 2 | **0.23 (0.07-0.75)** | 0.0%, 0.968 |  |  |  | 2 | 0.27 (0.06-1.17) | 0.0%,0.382 |
| *No priming with hCG* | 3 | **0.10 (0.02-0.59)** | 0.0%,0.747 |  |  |  | 2 | 0.20 (0.01-3.27) | 0.0%, 0.600 |
|  |  |  |  |  |  |  |  |  |  |
| Maturation  (oocytes-based analysis) | 8 | 0.87 (0.70-1.08) | 63.7%, 0.007 | 5 | **0.74 (0.59-0.93)** | 52.4%, 0.078 | 5 | 1.03 (0.88-1.21) | 0.0%, 0.734 |
| *Priming with hCG* | 4 | 0.82 (0.63-1.07) | 70.4%, 0.017 | 4 | **0.78 (0.62-0.98)** | 51.5%, 0.103 | 2 | 0.99 (0.81-1.22) | 0.0%, 0.406 |
| *No priming with hCG* | 4 | 0.94 (0.63-1.38) | 58.1%, 0.067 | 1 | **0.45 (0.23-0.86)** | NC | 3 | 1.10 (0.85-1.42) | 0.0%, 0.619 |
|  |  |  |  |  |  |  |  |  |  |
| Fertilization  (oocytes-based analysis) | 8 | 0.78 (0.60-1.03) | 71.3%, 0.001 | 5 | 0.76 (0.55-1.04) | 72.9%, 0.005 | 5 | 0.88 (0.54-1.43) | 79.9%, 0.001 |
| *Priming with hCG* | 3 | 0.74 (0.46-1.20) | 90.0%, <0.001 | 3 | 0.77 (0.51-1.18) | 83.9%, 0.002 | 2 | 0.84 (0.27-2.62) | 94.3%, <0.001 |
| *No priming with hCG* | 5 | 0.84 (0.65-1.10) | 0.0%, 0.556 | 2 | 0.71 (0.37-1.38) | 58.0%, 0.123 | 3 | 0.91 (0.65-1.28) | 0.0%, 0.925 |
|  |  |  |  |  |  |  |  |  |  |
| Miscarriage  (women-based analysis) | 4 | 1.51 (0.72-3.17) | 0.0%, 0.795 |  | Not applicable |  |  | Not applicable |  |
| *Priming with hCG* | 3 | 1.53 (0.72-3.27) | 0.0%, 0.606 |  |  |  |  |  |  |
| *No priming with hCG* | 1 | 1.19 (0.04-32.08) | NC |  |  |  |  |  |  |

^§^number of study arms, NC: not calculable

**Table D.** Subgroup analyses by ICSI / IVF procedure; the study by Soderstrom-Anttila, 2005 was subdivided into two separate study arms for this analysis. Only outcomes comprising four or more published studies are presented; bold cells denote statistically significant associations.

|  | PCOS vs. non-PCOS (PCO/healthy controls) | | | Subgroup analysis:  PCOS vs. healthy controls | | | Subgroup analysis:  PCOS vs. PCO | | |
| --- | --- | --- | --- | --- | --- | --- | --- | --- | --- |
|  | n^§^ | OR (95%CI) | Heterogeneity  I^2^, p | n^§^ | OR (95%CI) | Heterogeneity  I^2^, p | n^§^ | OR (95%CI) | Heterogeneity  I^2^, p |
| **Analysis stratified by stimulation with FSH** |  |  |  |  |  |  |  |  |  |
| Live birth  (cycles-based analysis) |  |  |  |  | Not applicable |  |  |  |  |
| *Study arms on ICSI* | 4 | 1.26 (0.65-2.42) | 0.0%, 0.819 |  |  |  | 4 | 0.95 (0.48-1.90) | 0.0%, 0.948 |
| *Study arms on IVF* | 1 | **3.21 (1.04-9.95)** | NC |  |  |  | 1 | 2.75 (0.46-16.59) | NC |
|  |  |  |  |  |  |  |  |  |  |
| Live birth  (women-based analysis) |  |  |  |  | Not applicable |  |  |  |  |
| *Study arms on ICSI* | 4 | 1.35 (0.69-2.61) | 0.0%, 0.769 |  |  |  | 4 | 1.02 (0.51-2.05) | 0.0%, 0.942 |
| *Study arms on IVF* | 1 | **3.21 (1.04-9.95)** | NC |  |  |  | 1 | 2.75 (0.46-16.59) | NC |
|  |  |  |  |  |  |  |  |  |  |
| Clinical pregnancy  (cycles-based analysis) |  |  |  |  |  |  |  |  |  |
| *Study arms on ICSI* | 6 | **1.92 (1.18-3.12)** | 0.0%, 0.643 | 4 | 2.66 (0.98-7.19) | 49.4%, 0.115 | 4 | 1.35 (0.72-2.53) | 0.0%, 0.876 |
| *Study arms on IVF* | 2 | **4.03 (1.49-10.93)** | 0.0%, 0.796 | 1 | **4.06 (1.41-11.68)** | NC | 2 | 4.69 (0.98-22.38) | 0.0%, 0.705 |
|  |  |  |  |  |  |  |  |  |  |
| Clinical pregnancy  (women-based analysis) |  |  |  |  |  |  |  |  |  |
| *Study arms on ICSI* | 6 | **2.06 (1.25-3.39)** | 0.0%, 0.532 | 4 | 2.83 (0.94-8.52) | 56.6%,0.075 | 4 | 1.49 (0.78-2.84) | 0.0%, 0.848 |
| *Study arms on IVF* | 2 | **4.03 (1.49-10.93)** | 0.0%, 0.796 | 1 | **4.06 (1.41-11.68)** | NC | 2 | 4.69 (0.98-22.38) | 0.0%, 0.705 |
|  |  |  |  |  |  |  |  |  |  |
| Cancellation  (cycles-based analysis) |  |  |  |  | Not applicable |  |  |  |  |
| *Study arms on ICSI* | 5 | **0.21 (0.07-0.67)** | 0.0%, 0.693 |  |  |  | 4 | 0.35 (0.07-1.69) | 0.0%, 0.854 |
| *Study arms on IVF* | 1 | **0.11 (0.11-0.83)** | NC |  |  |  | 1 | 0.13 (0.01-1.37) | NC |
|  |  |  |  |  |  |  |  |  |  |
| Maturation  (oocytes-based analysis) |  |  |  |  |  |  |  |  |  |
| *Study arms on ICSI* | 6 | 0.96 (0.74-1.24) | 66.6%, 0.010 | 4 | 0.84 (0.62-1.13) | 60.7%, 0.054 | 4 | 1.10 (0.91-1.33) | 0.0%, 0.820 |
| *Study arms on IVF* | 3 | **0.67 (0.46-0.99)** | 53.7%, 0.115 | 2 | **0.56 (0.43-0.74)** | 0.0%, 0.461 | 2 | 0.86 (0.63-1.19) | 0.0%, 0.438 |
|  |  |  |  |  |  |  |  |  |  |
| Fertilization  (oocytes-based analysis) |  |  |  |  |  |  |  |  |  |
| *Study arms on ICSI* | 6 | 0.81 (0.59-1.12) | 71.7%, 0.003 | 4 | 0.84 (0.57-1.24) | 74.9%, 0.008 | 4 | 0.75 (0.47-1.21) | 64.0%, 0.039 |
| *Study arms on IVF* | 3 | 0.93 (0.51-1.68) | 71.0%, 0.032 | 2 | 0.85 (0.31-2.38) | 85.0%, 0.010 | 2 | 1.22 (0.80-1.85) | 0.0%, 0.385 |
|  |  |  |  |  |  |  |  |  |  |
| Miscarriage  (women-based analysis) |  |  |  |  | Not applicable |  |  | Not applicable |  |
| *Study arms on ICSI* | 4 | 1.55 (0.68-3.55) | 0.0%, 0.672 |  |  |  |  |  |  |
| *Study arms on IVF* | 1 | 1.17 (0.22-6.28) | NC |  |  |  |  |  |  |
|  |  |  |  |  |  |  |  |  |  |

^§^number of study arms, NC: not calculable

**Table E.** Evaluation of quality based on the Newcastle-Ottawa scale for all eleven included studies.

|  | **Selection** | | | | **Comparability** | | **Outcome** | | | **Total** |
| --- | --- | --- | --- | --- | --- | --- | --- | --- | --- | --- |
| **Study** | Representativeness | Selection of non-exposed | Ascertainment of exposure | Outcome not present at start | On age | On other risk factors | Assessment of outcome | Long enough follow-up | Adequacy (completeness) of follow-up |  |
| Barnes (1996) | 1 | 1 | 1 | 1 | 0 | 0 | 1 | 0 | 0 | 5 |
| Buckett (2008) | 1 | 1 | 1 | 1 | 0 | 0 | 1 | 1 | 1 | 7 |
| Child (2001) | 1 | 1 | 1 | 1 | 1 | 1 | 1 | 1 | 0 | 8 |
| de Vos (2011) | 1 | 1 | 1 | 1 | 1 | 0 | 1 | 1 | 1 | 8 |
| Junk (2012) | 1 | 1 | 1 | 1 | 1 | 1 | 1 | 1 | 1 | 9 |
| Kedem (2013a) | 1 | 1 | 1 | 1 | 1 | 1 | 1 | 0 | 0 | 7 |
| Mikkelsen (2001) | 1 | 1 | 1 | 1 | 0 | 0 | 1 | 0 | 0 | 5 |
| Soderstrom-Anttila (2005) | 1 | 1 | 1 | 1 | 1 | 1 | 1 | 1 | 1 | 9 |
| Trounson, exp 2 (1994) | 1 | 1 | 1 | 1 | 0 | 0 | 1 | 0 | 0 | 5 |
| Vieira (2011) | 1 | 1 | 1 | 1 | 1 | 1 | 1 | 0 | 0 | 7 |
| Zhao (2006) | 1 | 1 | 1 | 1 | 1 | 1 | 1 | 1 | 0 | 8 |
